# Downregulation of UBE4B promotes CNS axon regrowth and functional recovery after stroke

**DOI:** 10.1016/j.isci.2022.105885

**Published:** 2022-12-26

**Authors:** Shuang Jin, Xiangfeng Chen, Hanyu Zheng, Wanxiong Cai, Xurong Lin, Xiangxing Kong, Yingchun Ni, Jingjia Ye, Xiaodan Li, Luoan Shen, Binjie Guo, Zeinab Abdelrahman, Songlin Zhou, Susu Mao, Yaxian Wang, Chun Yao, Xiaosong Gu, Bin Yu, Zhiping Wang, Xuhua Wang

**Affiliations:** 1 Department of Neurobiology and Department of Rehabilitation Medicine, First Affiliated Hospital, Zhejiang University School of Medicine, Hangzhou, Zhejiang Province 310003, P.R. China; 2 Liangzhu Laboratory, MOE Frontier Science Center for Brain Science and Brain-Machine Integration, State Key Laboratory of Brain-Machine Intelligence, Zhejiang University, 1369 West Wenyi Road, Hangzhou 311121, P.R. China; 3 NHC and CAMS Key Laboratory of Medical Neurobiology, Zhejiang University, Hangzhou 310058, P.R. China; 4 Co-Innovation Center of Neuroregeneration, Nantong University, Nantong, Jiangsu 226001, P.R. China; 5 Department of Neurobiology and Department of Orthopedics, 2nd Affiliated Hospital, Zhejiang University School of Medicine, Hangzhou, Zhejiang Province 310003, P.R. China; 6 Key Laboratory of Neuroregeneration of Jiangsu and Ministry of Education, Nantong University, Nantong, Jiangsu 226001, P.R. China

**Keywords:** Biological sciences, Neuroscience, Molecular neuroscience

## Abstract

The limited intrinsic regrowth capacity of corticospinal axons impedes functional recovery after cortical stroke. Although the mammalian target of rapamycin (mTOR) and p53 pathways have been identified as the key intrinsic pathways regulating CNS axon regrowth, little is known about the key upstream regulatory mechanism by which these two major pathways control CNS axon regrowth. By screening genes that regulate ubiquitin-mediated degradation of the p53 proteins in mice, we found that ubiquitination factor E4B (UBE4B) represses axonal regrowth in retinal ganglion cells and corticospinal neurons. We found that axonal regrowth induced by UBE4B depletion depended on the cooperative activation of p53 and mTOR. Importantly, overexpression of UbV.E4B, a competitive inhibitor of UBE4B, in corticospinal neurons promoted corticospinal axon sprouting and facilitated the recovery of corticospinal axon-dependent function in a cortical stroke model. Thus, our findings provide a translatable strategy for restoring corticospinal tract-dependent functions after cortical stroke.

## Highlights

• Deregulation of UBE4B is sufficient in promoting CNS axon regrowth
• UBE4B regulates CNS axon regrowth through p53 and KLHL22/mTOR dual pathways
• Modulation of UBE4B pathway via UbV.E4B overexpression promotes CNS axon regrowth
• CST sprouting induced by UbV.E4B mediates CST-dependent function recovery after stroke

## Graphical abstract

**Figure:**
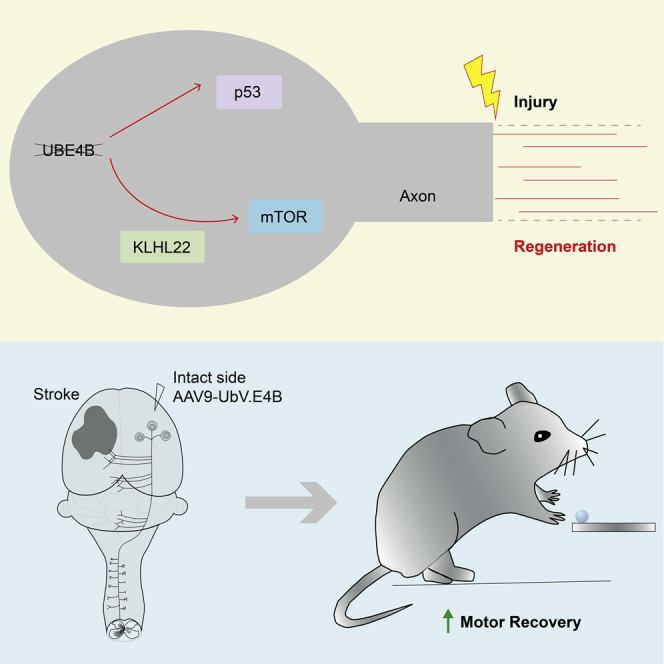

## Introduction

Promoting the regrowth of corticospinal tract (CST) axons to reinnervate the spinal cord is a promising strategy for restoring lost functions after cortical stroke.^1^^,^^2^ However, CST axons in the adult CNS are intrinsically refractory to regrowth after cortical stroke. An understanding of the basic biological processes within neurons that intrinsically inhibit axonal regrowth is a prerequisite for developing interventions for stroke or CNS injury. In the past decade, numerous intrinsic factors that regulate the regrowth capacity of CNS axons have been identified.^3^^,^^4^^,^^5^^,^^6^^,^^7^ Studies have shown that increasing the activity of mammalian target of rapamycin (mTOR) markedly augments the axonal regeneration capacity of injured neurons.^4^^,^^5^^,^^8^^,^^9^^,^^10^ Moreover, the p53 pathway has been reported to regulate the intrinsic axonal regrowth capacity of CNS neurons.^11^^,^^12^ However, little is known about the key upstream regulatory mechanism by which the p53 pathway and mTOR pathway control CNS axon regrowth.

Ubiquitin pathways, which participate in the protein quality control system, are responsible for maintaining cellular homeostasis by degrading unfolded and misfolded proteins. After axonal injury, damaged proteins must be quickly eliminated to help restore cellular homeostasis for axonal regrowth. Therefore, ubiquitin pathways have been suggested to play important roles in this process.^13^^,^^14^ However, how and to what extent ubiquitin pathways regulate CNS axon regrowth remain unclear. Because ubiquitin pathways have been reported to regulate axonal regrowth through the p53 axis,^11^ we speculate that ubiquitin-mediated degradation of p53 might play an important role in controlling CNS axon regrowth.

We screened a series of genes involved in regulating ubiquitination of p53 to test this hypothesis and identified the ubiquitination factor E4B (UBE4B), an E3 and E4 ubiquitin ligase, as a new repressor of CNS axon regrowth. Surprisingly, we found that UBE4B also played a key role in modulating the mTOR pathway, another major regeneration-promoting pathway,^4^^,^^15^ via its substrate KLHL22. In addition, overexpression of the ubiquitin variant UbV.E4B, which was previously designed as an effective inhibitor of UBE4B activity,^16^ in corticospinal neurons led to robust CST axon sprouting and the recovery of CST axon-dependent functions in a rat cortical stroke model. Thus, our findings not only reveal the key mechanism that simultaneously regulates the two major CNS regeneration regulation pathways but also provide a translatable strategy for restoring CST axon-dependent functions after cortical stroke.

## Results

### Identification of UBE4B as a key inhibitor of CNS axon regrowth

**Figure 1:**
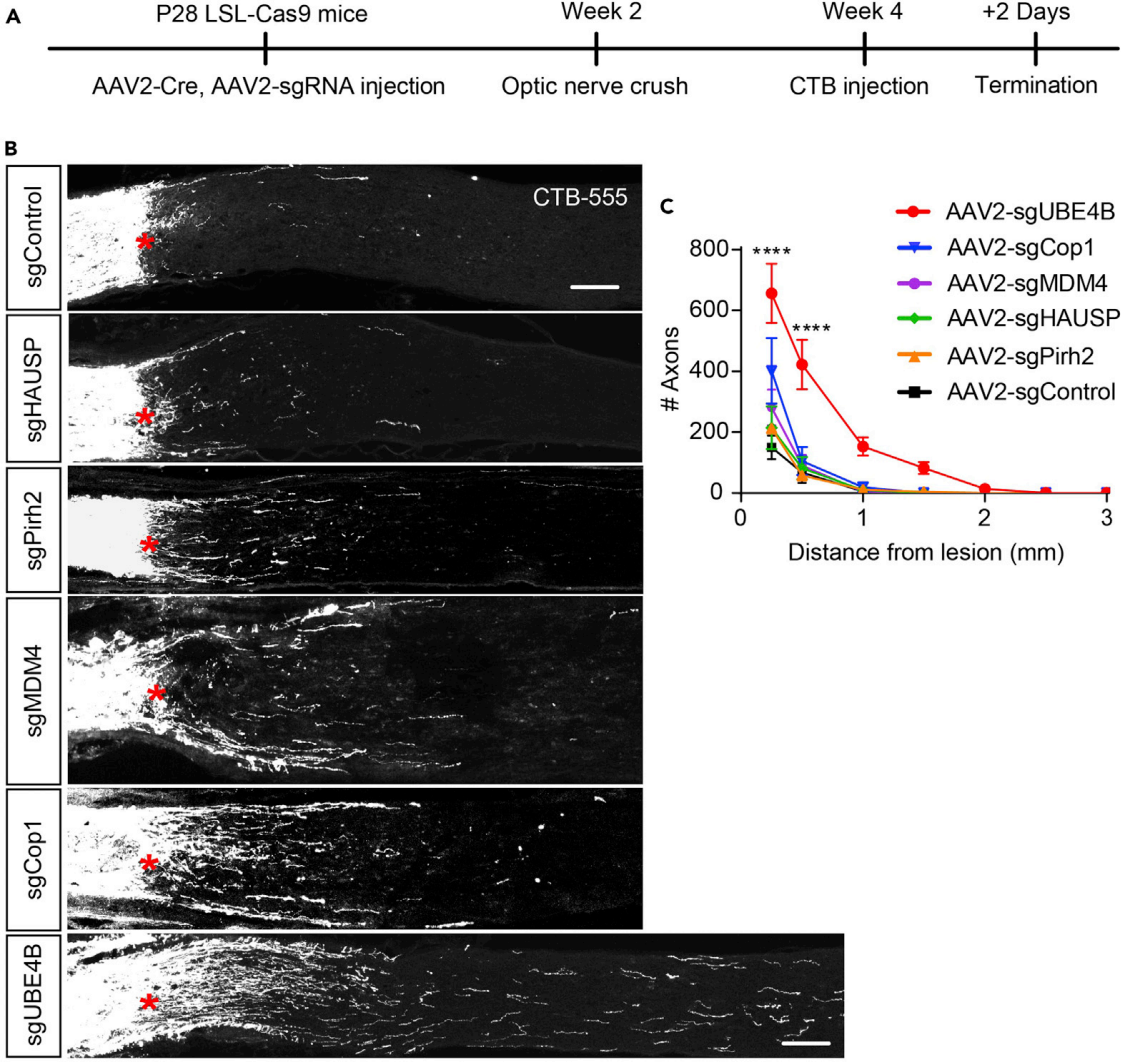
Identification of UBE4B as a key inhibitor of CNS axon regeneration (A) Timeline of the experimental procedure used to study optic nerve regeneration. (B) Representative images of optic nerve sections showing CTB-labeled axons in LSL-Cas9 mice with gene knockout induced by an intravitreal injection of AAV2-Control-sgRNA, AAV2-UBE4B-sgRNA, AAV2-Pirh2-sgRNA, AAV2-Cop1-sgRNA, AAV2-HAUSP-sgRNA or AAV2-MDM4-sgRNA, and AAV2-Cre at 2 weeks after optic nerve injury. The crush site is indicated by a red asterisk. The scale bar represents 100 μm. (C) Quantification of the regenerating axons in (B). The data are presented as the means ± SEM (n = 3–9). ∗∗∗∗p < 0.0001 (ANOVA with Bonferroni’s post hoc test, compared to the AAV2-sgControl group).

We screened a series of ubiquitin-related genes involved in p53 degradation, including HAUSP, Pirh2, COP1, MDM4, and UBE4B, to identify the key ubiquitin-related molecule that regulates axonal regrowth via the p53 pathway.^11^^,^^17^^,^^18^^,^^19^^,^^20^^,^^21^ In a pilot experiment, we employed an optic nerve crush (ONC) model to investigate the roles of these genes in regulating CNS axon regeneration because the anatomical structure of the optic nerve is relatively simple and because findings obtained using optic nerve injury models have been verified in other models of axonal injury.^6^ Adeno-associated virus (AAV) serotype 2 (AAV2) vectors carrying single-guide RNA (sgRNA) and cyclization recombination enzyme (Cre) were intravitreally injected into the retinas of Rosa26-Loxp-Stop-Loxp-Cas9 knockin mice (LSL-Cas9 mice) to delete the target genes via CRISPR/Cas9 technology,^22^^,^^23^ and the gene knockout efficiency obtained using this method was validated (Tables S1 and S2). We also coadministered AAV2-sgControl and AAV2-Cre into control LSL-Cas9 mice by intravitreal injection. Two weeks after virus injection, ONC was performed using a well-established protocol.^4^ After another 2 weeks, axon regeneration was evaluated by injecting the anterograde fluorescent tracer-conjugated cholera toxin subunit B (CTB) into the vitreous humor 2 days prior to tissue collection (Figure 1A).

As a result, knockout of MDM4 in retinal ganglion cells (RGCs) promoted optic nerve axon regeneration to some extent, i.e., to approximately the same extent reported in a previous study (Figure 1B).^11^ Interestingly, knockout of COP1 also promoted axonal regeneration to some extent, promoting regeneration of a limited number of axons over a limited distance (Figure 1B). However, after knockout of other factors, such as Pirh2 and HAUSP, which have also been proven to be involved in ubiquitin pathways, almost no or few regenerating axons were observed after injury, similar to results observed in control mice (Figures 1B and 1C), although these proteins are expressed in the adult CNS (proteinatlas.org).^24^^,^^25^^,^^26^^,^^27^^,^^28^ In addition, RGC survival was not affected by knockout of these genes individually (data not shown). Besides, we found the deletion of MDM2, a previously reported gene,^11^ leads to some axon regeneration after optic nerve injury (data not shown). Interestingly, we found that only UBE4B knockout promoted abundant RGC axon regeneration after injury and that the regenerated axons extended approximately 1.5 mm from the lesion (Figure 1B), possibly because UBE4B knockout leads to the activation of other pathways mediating axon regeneration in addition to the p53 pathway. Moreover, we knocked out both MDM2 and UBE4B in UBE4B^f/f^-LSL-Cas9 mice via intravitreal injections of AAV2-Cre and AAV2-U6-sgMDM2. Interestingly, the deletion of both UBE4B and MDM2 leads to more robust regeneration than the deletion of UBE4B or MDM2 alone without affecting the survival rate of RGCs (data not shown).

**Figure 2:**
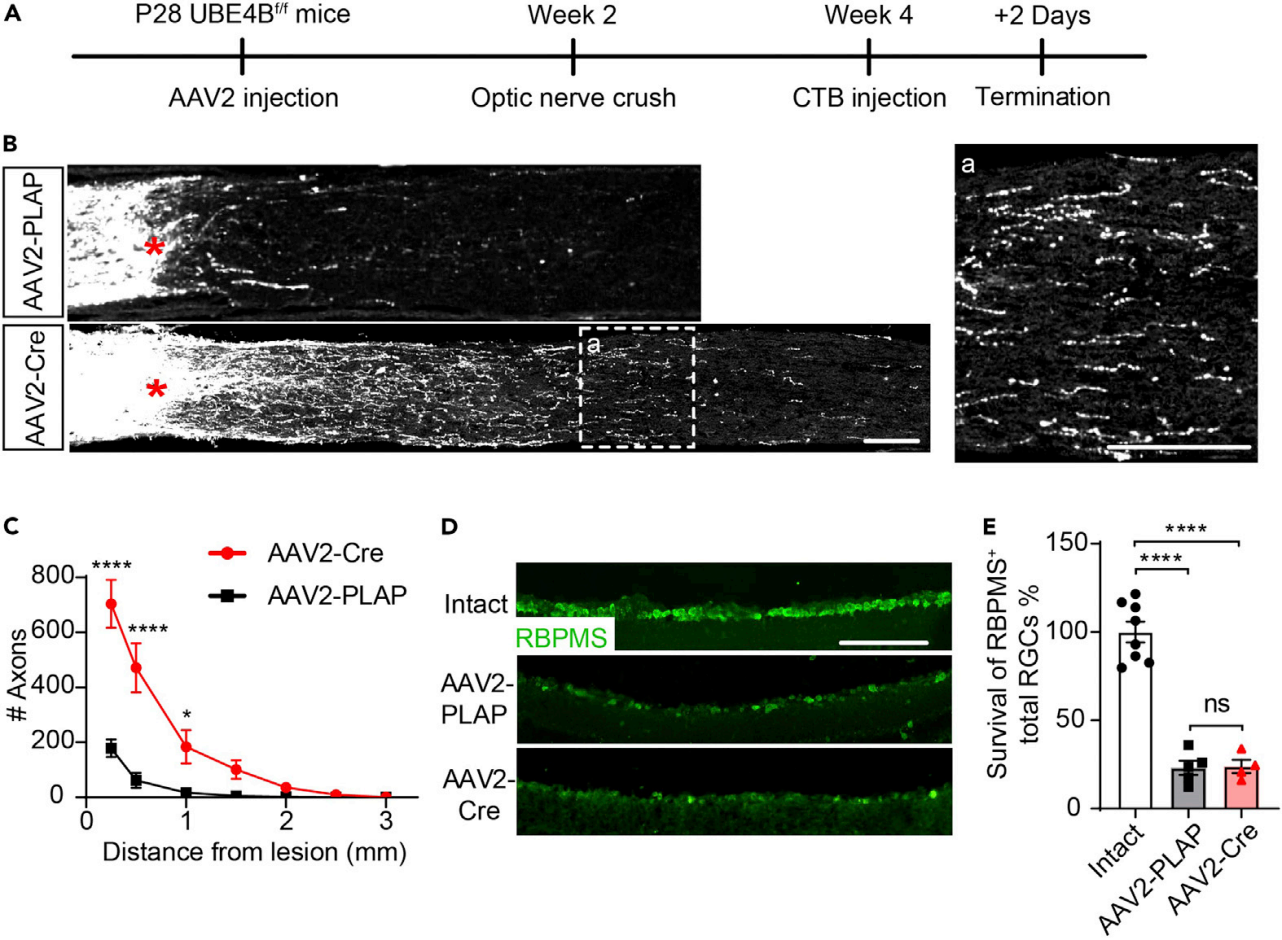
UBE4B deficiency promotes optic nerve regeneration (A) Timeline of the experimental procedure used to study optic nerve regeneration. (B) Representative images of optic nerve sections showing CTB-labeled axons in UBE4B^f/f^ mice that received intravitreal injections of AAV2-PLAP (AAV2-Control) and AAV2-Cre at 2 weeks after optic nerve injury. The crush site is indicated by a red asterisk. (a) Enlarged image of axons 1,000 μm from the lesion site. The scale bars in (B) and (a) represent 100 μm. (C) Quantification of regenerating axons in (B) at different distances from the injury site (n = 4–5). The data are presented as the means ± SEM ∗p < 0.05 and ∗∗∗∗p < 0.0001 (ANOVA with Bonferroni’s post hoc test). (D) Representative images of RBPMS staining in sections of intact retinas or injured retinas 2 weeks after injury following the injection of AAV2-PLAP or AAV2-Cre. The scale bar represents 100 μm. (E) Quantification of the data in (D). The data are presented as the means ± SEM (n = 4–8). ∗∗∗∗p < 0.0001 (ANOVA with Bonferroni’s post hoc test).

Given the limited knockout efficiency and potential off-target effects of the CRISPR/Cas9 technique, we generated homozygous conditional UBE4B-knockout (UBE4B^f/f^) mice to further verify the effect of UBE4B knockout on axonal regeneration (Figure 2A). AAV2-Cre or AAV2-PLAP control was intravitreally injected into UBE4B^f/f^ mice, and the regeneration of optic nerve axons was significantly increased in the group in which UBE4B was deleted in RGCs compared with the control group (Figures 2A–2C), consistent with the results obtained using the CRISPR/Cas9 technique. Because gene knockout might also affect RGC survival, we assessed the RGC survival rate 2 weeks after ONC injury in AAV2-PLAP-injected and UBE4B-knockout mice with the pan-RGC marker RNA binding protein with multiple splicing (RBPMS)^29^ and retinal whole-mount staining for Tuj1.^4^^,^^9^ The RGC survival rate was not affected by UBE4B knockout (Figures 2D, 2E, S1A, and S1B), indicating that UBE4B knockout promotes optic nerve regeneration by enhancing the regeneration potential of surviving RGCs after ONC rather than by protecting against RGC death after ONC.

### UBE4B deletion promotes optic nerve regeneration via both the p53 and mTOR pathways

As heterozygosity of UBE4B is sufficient to induce cellular stress in at least some types of neurons^30^ and that stress signaling is an important component of the regenerative response,^31^ we first sought to investigate whether UBE4B knockout primes RGCs for more robust axon regeneration by activating stress response pathways prior to injury. Immunohistochemical staining for the transcription factors ATF3 and c-Jun was conducted to clarify whether this possible mechanism might contribute to the regenerative phenotypes observed in UBE4B-knockout animals (Figures S2A and S2C). We immunostained retinal sections collected three weeks after the intravitreal injection of AAV2-Cre or AAV2-PLAP control into UBE4B^f/f^ mice. Our results indicate that UBE4B deletion did not increase the expression of ATF3 (Figures S2A and S2B) or c-Jun (Figures S2C and S2D), suggesting that UBE4B knockout does not activate stress response pathways prior to injury.

**Figure 3:**
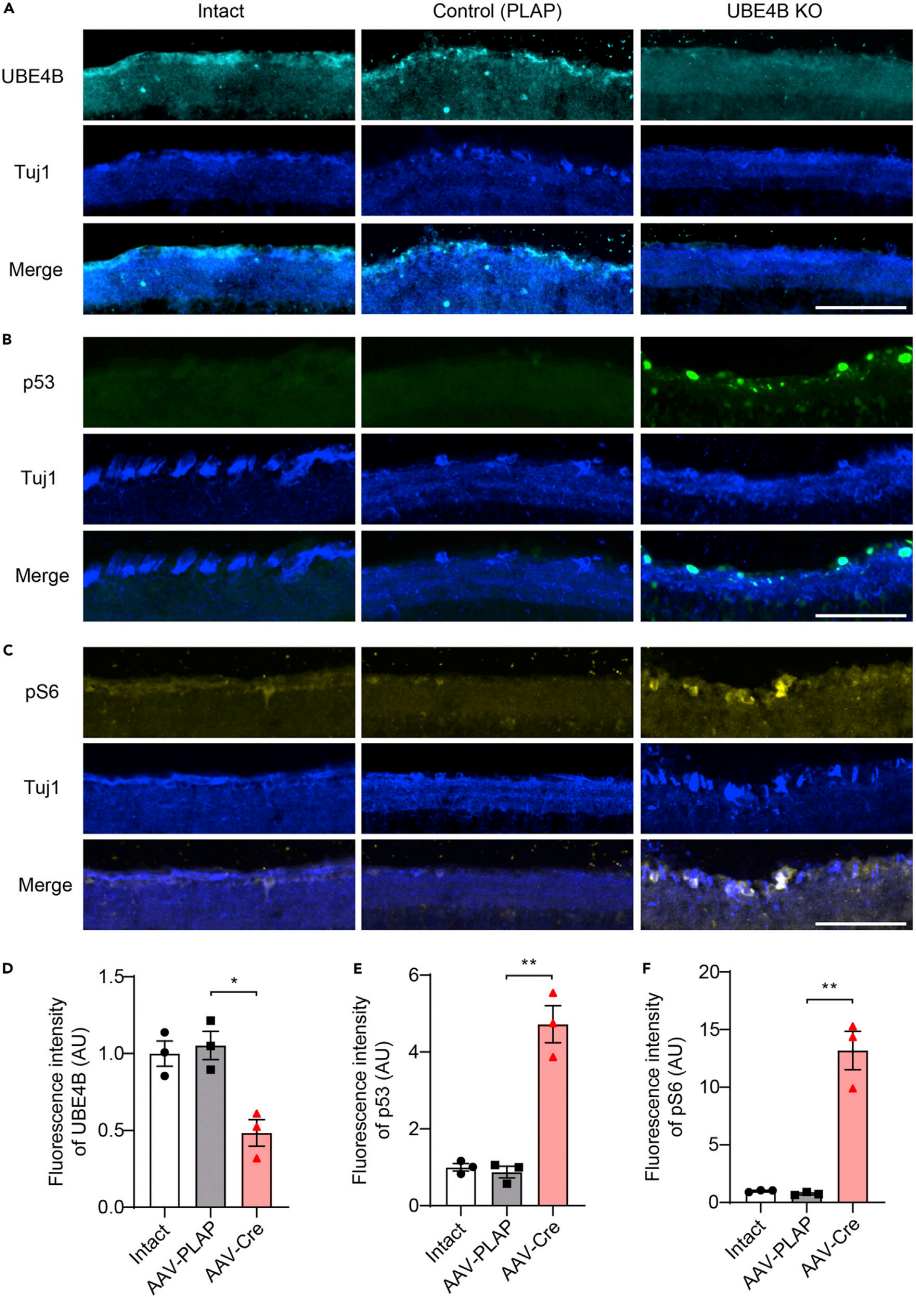
UBE4B deletion upregulates p53 and mTOR expression (A–C) Immunofluorescence staining for UBE4B (A), p53 (B), and pS6 (C) in sections of intact retinas and the retinas of UBE4B^f/f^ mice injected with AAV2-Control or AAV2-Cre 2 weeks after crush injury. The scale bar represents 100 μm. (D–F) Quantification of the fluorescence intensity of UBE4B (D), p53 (E), and pS6 (F) in (A, B, and C). At least three nonconsecutive sections of the ganglion cell layer of retinas from three or four mice per group were used for quantification. The data are presented as the means ± SEM ∗p < 0.05 and ∗∗p < 0.01 (Student’s *t* test).

The mTOR pathway is a key pathway regulating axonal regeneration,^4^^,^^15^ and we speculated that UBE4B deletion might promote axonal regrowth by altering the activity of mTOR in addition to that of p53. We immunostained retinal sections collected two weeks after ONC injury for UBE4B, p53, and pS6, an indicator of mTOR activity, to test this hypothesis. As expected, we observed the significant downregulation of UBE4B expression (Figures 3A and 3D) and upregulation of p53 expression (Figures 3B and 3E) in UBE4B-knockout RGCs. Strikingly, UBE4B knockout significantly upregulated pS6 expression after ONC (Figures 3C and 3F), indicating that UBE4B regulated both the p53 pathway and mTOR pathway after ONC injury. In a companion paper, Kong et al. performed proteomic analysis of UBE4B-knockout cortical neurons and identified KLHL22 as a major substrate of UBE4B.^32^ Interestingly, a previous study showed that the KLHL22 ubiquitin ligase complex plays a conserved role in mediating the activation of mTORC1 and downstream events in mammals, and the KLHL22/mTOR axis was proven to activate amino acid-dependent mTORC1 signaling.^33^ Therefore, we speculated that UBE4B deletion might increase the regeneration potential of RGCs via the KLHL22/mTOR pathway.

**Figure 4:**
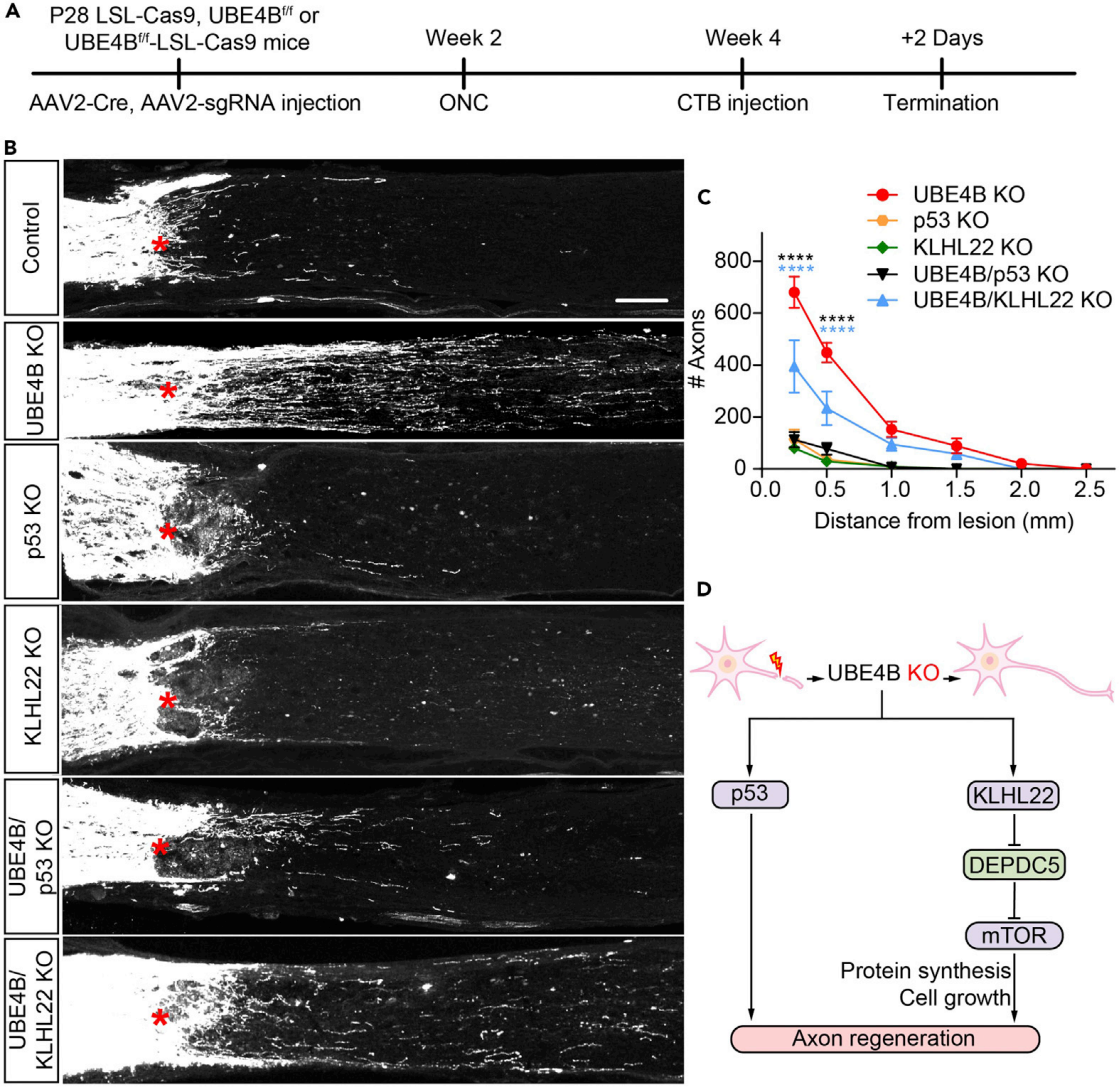
UBE4B knockout mediates axonal regeneration by modulating two pathways (A) Time course of regeneration experiments involving conditional knockout of UBE4B and/or p53/KLHL22. (B) Confocal images of optic nerves from UBE4B, p53, and KLHL22 knockout or UBE4B/p53, UBE4B/KLHL22 double-knockout mice showing CTB-labeled axons around the lesion sites 2 weeks after injury. The crush site is indicated by a red asterisk. The scale bar represents 100 μm. (C) Quantification of regenerating axons at different distances distal to the lesion sites 2 weeks after crush injury in (B). The data are presented as the means ± SEM (n = 3–5). ∗∗∗p < 0.001 and ∗∗∗∗p < 0.0001 (ANOVA with Bonferroni’s post hoc test, compared to the control group). (D) Schematic of the UBE4B/p53 and UBE4B/KLHL22/mTOR signaling pathways.

We directly investigated the contributions of p53- or KLHL22-dependent pathways to the effects of UBE4B deletion on axonal regeneration by crossing UBE4B^f/f^ mice with LSL-Cas9 mice to generate homogeneous mutants carrying two floxed genes (UBE4B and Cas9). The double-mutant mice received an intravitreal viral injection of AAV2-Cre and/or AAV2-sgRNA to induce the deletion of one or both genes in RGCs (Figure 4A). The regeneration of injured optic nerve axons was not significantly different between the p53- or KLHL22-knockout mice and the control mice (Figures 4B and 4C). However, additional knockout of p53 or KLHL22 led to a significant decrease in axonal regeneration in UBE4B-knockout mice (Figures 4B and 4C), indicating that p53 and KLHL22 deletion partially inhibited axonal regeneration triggered by UBE4B deletion. Moreover, no significant differences in neuronal survival were observed among the UBE4B-knockout, p53-knockout, KLHL22-knockout groups, and the double-knockout groups (Figures S3A and S3B), suggesting that the apoptosis of RGCs does not impair axonal regeneration. Furthermore, combining AAV2-Cre and AAV2-sgRNA (for p53 or KLHL22) with AAV expressing ciliary neurotrophic factor (AAV-CNTF) did not affect axonal regeneration induced by CNTF (Figures S4A–S4C). P53 knockout and KLHL22 knockout did not affect the survival rate of RGCs promoted by CNTF overexpression after injury (Figures S4D and S4E). Conclusively, we confirmed that UBE4B deletion in RGCs dramatically promoted axonal regeneration following optic nerve injury through a mechanism depending on the p53 and KLHL22/mTOR pathways (Figure 4D).

### UBE4B and PTEN double knockout further promotes optic nerve regeneration

**Figure 5:**
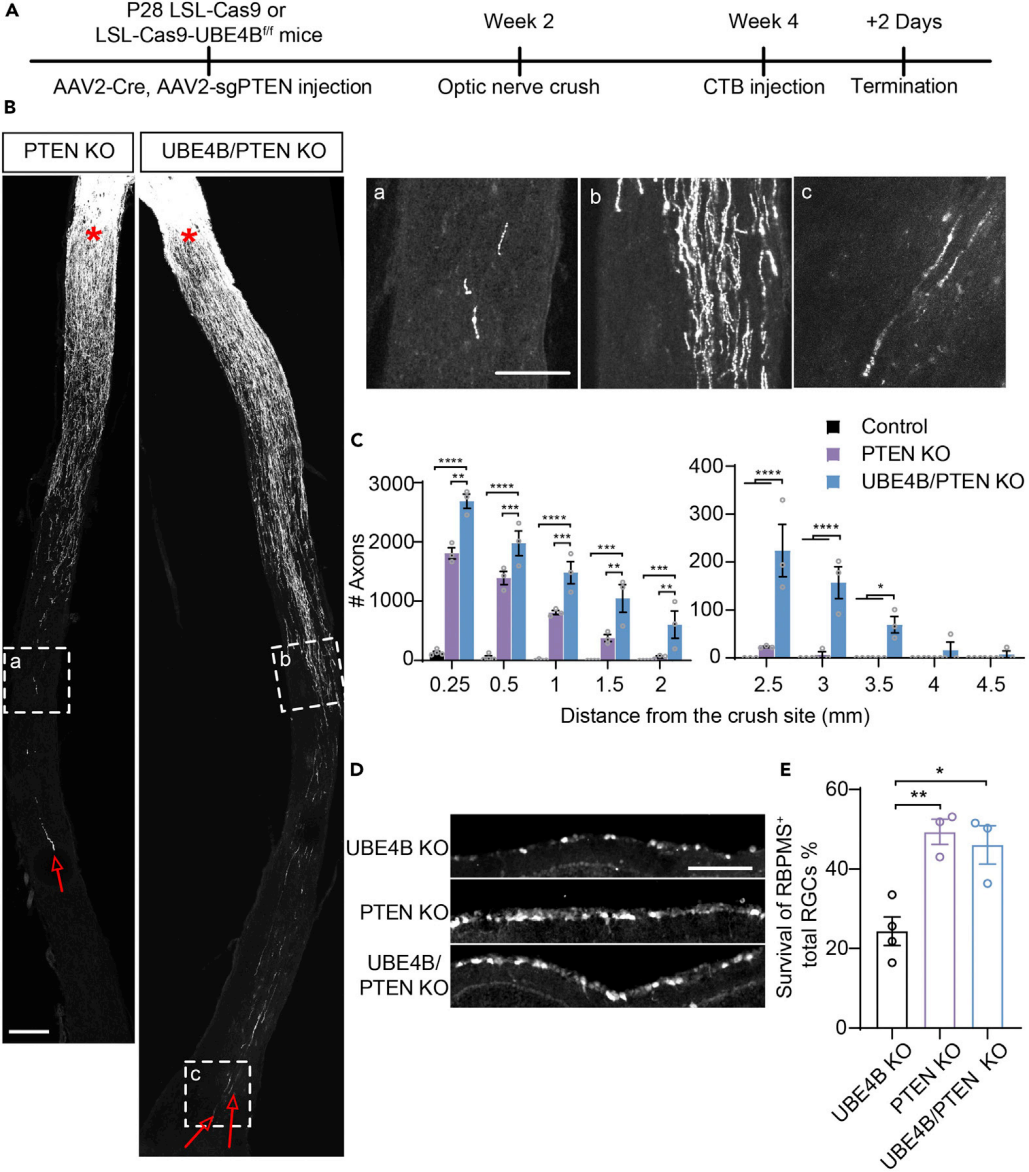
UBE4B and PTEN double knockout further promotes optic nerve regeneration (A) Timeline of the experimental procedure used to study optic nerve regeneration. (B) Representative images showing that the deletion of both PTEN and UBE4B in RGCs induced faster axonal regeneration in optic nerves 2 weeks after ONC. The red asterisk indicates the crush sites. The red arrows indicate the longest axons of each nerve. The scale bar represents 200 μm. High magnification images of the boxed area in (B), i.e., nerves at 2,500 μm (a, b) and 4,500 μm (c) from the lesion site, are shown. The scale bar represents 100 μm. (C) Quantification of regenerating axons at different distances from the injury site shown in (B). ANOVA followed by Bonferroni’s multiple comparisons test (n = 3–4). ∗p < 0.05, ∗∗p < 0.01, ∗∗∗p < 0.001, and ∗∗∗∗p < 0.0001. The data are presented as the means ± SEM. (D) Representative experimental retinal sections stained with anti-RBPMS antibodies 2 weeks after injury following the injection of AAV2-Cre and/or AAV2-sgPTEN. The scale bar represents 100 μm. (E) Quantification of the data in (D). RBPMS-positive cells in the ganglion cell layer were imaged. The data are presented as the means ± SEM (n = 3–4). ∗p < 0.05 and ∗∗p < 0.01 (ANOVA with Bonferroni’s post hoc test).

Previous studies have indicated that modulation of the PTEN/mTOR pathway increases the survival of RGCs and promotes axon regeneration.^4^^,^^5^^,^^34^^,^^35^ Our finding that the effect of UBE4B deletion also involves the mTOR pathway motivated us to explore the synergistic effect of UBE4B and PTEN deletion on axonal regeneration. In Rosa26-LSL-Cas9 mice, we deleted PTEN using AAV2-Cre mixed with AAV2-sgPTEN (PTEN-knockout group). In UBE4B^f/f^-LSL-Cas9 mice, we knocked out both PTEN and UBE4B by administering intravitreal injections of AAV2-Cre and AAV2-U6-sgPTEN (UBE4B/PTEN-knockout group) (Figure 5A). We found that double knockout of UBE4B and PTEN induced optic nerve regeneration to a greater extent than PTEN knockout alone (Figures 5B, 5a, 5b and 5c). Specifically, compared with UBE4B knockout alone and PTEN knockout alone, knockout of both UBE4B and PTEN induced more axonal regeneration at 500, 750, 1,000, 1,500, 2,000, 2,500, 3,000, and 3,500 μm from the injury site, with many axons reaching the optic chiasm within 2 weeks after injury, which was rare after knockout of either gene alone (Figures 2C and 5C). Notably, the survival of RBPMS^+^ RGCs increased to approximately 50% in UBE4B/PTEN double-knockout mice (Figures 5D and 5E). Based on these results, the deletion of both UBE4B and PTEN led to more robust regeneration than the deletion of UBE4B or PTEN alone, possibly because UBE4B knockout promoted the activation of the p53 pathway in addition to the mTOR pathway, further increasing the axonal regeneration capacity of RGCs after ONC injury.

### UBE4B knockout promotes CST axon sprouting in the spinal cord after unilateral cortical stroke

After verifying that UBE4B deletion in RGCs promotes RGC axon regeneration after injury, we attempted to assess whether this phenomenon also occurred in cortical neurons. Before the CST axon regrowth study, we first sought to assess whether the molecular pathways found to be involved in axonal regeneration in RGCs were also involved in axon regeneration in UBE4B-knockout corticospinal neurons. Therefore, we crossed UBE4B^f/f^ mice with UBE4B^f/f^ and Camk2α-Cre/+ mice to generate double-mutant mice in which UBE4B was specifically knocked out in neurons expressing Camk2α (Figure S5A). Immunoblot analyses of these cortical neurons confirmed that UBE4B deletion upregulated the expression of p53, KLHL22, pS6, and mTOR (Figures S5B and S5C), consistent with the alterations in RGCs induced by UBE4B knockout. This result confirmed that UBE4B knockout also regulated both the p53 pathway and mTOR pathway in cortical neurons.

We next sought to assess whether UBE4B deletion promoted CST axon regrowth in a cortical stroke model. We first injected AAV9-Cre (UBE4B-knockout group) or AAV9-PLAP (control) into the right sensorimotor cortices of neonatal UBE4B^f/f^ mice (Figures S6A and S6B). Six weeks after the virus injection, unilateral cortical stroke in the left sensorimotor cortex was elicited by generating a minimally invasive and reproducible photochemical cortical lesion to disrupt CST axons (Figures S6A and S6B). Six weeks after unilateral cortical stroke, AAV9-mCherry was injected into the intact sensorimotor cortices of the mice, and axons in the intact sensorimotor cortex were labeled with mCherry before sacrifice. The difference in injury-induced CST axon sprouting between control and UBE4B-knockout mice was evaluated by constructing color-coded heatmaps showing the density of CST sprouting in the contralateral denervated spinal hemicord. Although limited sprouting was observed in the control mice, the sensorimotor cortices of UBE4B-knockout mice showed increased sprouting into the ipsilateral spinal cord at the cervical level in Mid, Z1, and Z2 (Figure S6C). More axons extended into D1, D2, and D3 through the midline in the UBE4B-knockout mice (Figure S6D). Thus, the deletion of UBE4B strikingly enhanced the sprouting of adult CST axons following cortical stroke.

### Overexpression of UbV.E4B in corticospinal neurons improves functional recovery in a rat cortical stroke model by promoting CST axon sprouting

The UBE4B knockout-induced increase in CST axon sprouting inspired us to assess whether this axonal plasticity contributed to functional recovery after cortical stroke.^36^ Because the clinical application of UBE4B knockout strategies is limited, we sought to overexpress UbV.E4B, a ubiquitin variant-based inhibitor of UBE4B, and subsequently inhibit the activity of UBE4B (the sequences of UbV.E4B and UbiquitinWT are provided in Table S3).^16^ Before this experiment, we first attempted to test the effect of UbV.E4B overexpression in RGCs on optic nerve axon regrowth. For this experiment, we intravitreally injected AAV2-PLAP, AAV2-HA-UbiquitinWT, or AAV2-HA-UbV.E4B into the wild-type mice (Figure S7A). Unlike the overexpression of HA-UbiquitinWT or PLAP, overexpression of UbV.E4B in mature RGCs promoted robust optic nerve regeneration after ONC (Figures S7B–S7D). We next examined whether UbV.E4B functioned in RGCs. As UBE4B promotes p53 polyubiquitination and degradation,^19^ we used immunohistochemistry to analyze the expression of p53 in the RGC layer of retinal sections. Our data showed that HA-labeled UbV.E4B was overexpressed (Figures S7D and S7E) and reduced the degradation of p53 in RGCs (Figures S7F and S7G), indicating the inhibitory effect of UbV.E4B on UBE4B.

**Figure 6:**
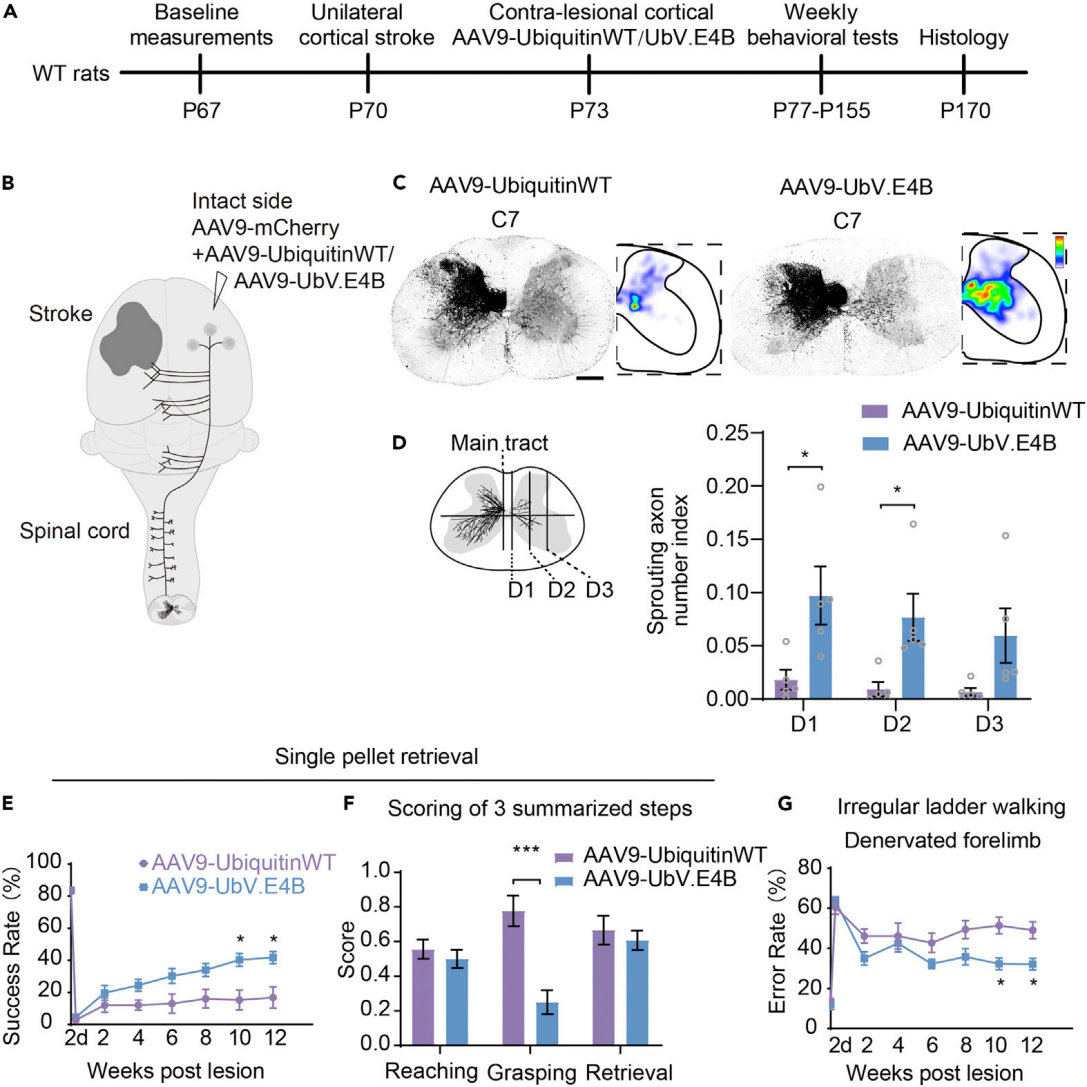
UbV.E4B treatment oromotes CST axon sprouting in the spinal cord and improves skilled locomotor ability after unilateral cortical stroke (A) Schematic diagram of the experimental timeline. Wild-type rats underwent baseline behavioral training at P67, unilateral cortical photothrombotic stroke at P70, unilateral cortical injection (the intact side) of AAV9-UbiquitinWT or AAV9-UbV.E4B at P73, and biweekly behavioral testing from P77 to P155 before the terminal histological analysis. (B) Schematic of the experimental procedure. Cortical injections (intact side) of AAV9-UbiquitinWT or AAV9-UbV.E4B and AAV9-mCherry were performed 3 days after unilateral photothrombotic stroke. Collateral sprouting of corticospinal axons occurred in the spinal cord of the control (AAV9-UbiquitinWT) and experimental (AAV9-UbV.E4B) groups after injury. (C) Representative images of transverse sections of spinal cords (C7) from AAV9-UbiquitinWT- and AAV9-UbV.E4B-treated animals stained with an anti-RFP antibody. The distribution of axonal sprouting on the denervated side was visualized in heatmaps; red represents the highest numbers of axon pixels, blue represents the lowest numbers of axon pixels, and white represents the background. The scale bar represents 500 μm. (D) Quantification of midline-crossing axons in different regions of the cervical spinal cord (C7) in AAV9-UbiquitinWT- and AAV9-UbV.E4B-injected groups. The schematic on the left illustrates the division of different regions of the spinal cord. D1, D2, and D3 represent different lateral positions. ∗p < 0.05 (Student’s *t* test; n = 5 mice per group). Three or four sections of the C7 spinal cord were quantified per mouse. The data are presented as the means ± SEM. (E) Performance on the single-pellet retrieval task. ∗p < 0.05 (repeated-measures ANOVA with Bonferroni’s post hoc correction; n = 10 AAV9-UbiquitinWT-injected animals; n = 19 AAV9-UbV.E4B-injected animals). (F) The scores for the three motor components in the behavioral tests. ∗∗∗p < 0.001 (one-way ANOVA followed by post hoc Student’s test; n = 9 AAV9-UbiquitinWT-injected rats; n = 14 AAV9-UbV.E4B-injected rats). (G) Forelimb function in the irregular ladder walking task. ∗p < 0.05 (repeated-measures ANOVA with Bonferroni’s post hoc correction; n = 9 AAV9-UbiquitinWT-injected animals; n = 15 AAV9-UbV.E4B-injected animals). The data are presented as the means ± SEM in (E), (F) and (G).

After confirming the ability of UbV.E4B overexpression to promote optic nerve regeneration after ONC, we next sought to assess whether this strategy also promoted CST axon regrowth and related functional recovery after cortical stroke. For this experiment, adult rats were first trained to master a single-pellet grasping task and irregular ladder walking task (Figure 6A) as the CST is known to be crucial for dexterous movements.^1^^,^^37^ Then, we disrupted CST axons on one side by inducing cortical stroke, and both triphenyltetrazolium chloride (TTC) staining (1 day after stroke) and protein kinase C gamma (PKCγ) staining in the cervical spinal cord (12 weeks after stroke) were conducted to validate the damage to the sensorimotor cortex (Figures S8A–S8C). We detected approximately 10% spared CST axons from the impacted cortex compared to the uninjured side (Figure S8D), suggesting that our rat cortical stroke model resulted in consistent lesions of the sensorimotor cortex. AAV9-HA-UbV.E4B was injected into the sensorimotor cortex on the intact side 3 days after stroke (Figure 6A). AAV9-UbiqutinWT was injected as a control (Figure 6B). The difference in injury-induced sprouting between control- and UbV.E4B-treated rats was evaluated by constructing color-coded heatmaps showing the density of CST sprouting in the contralateral denervated spinal hemicord (Figure 6C). We found that HA-UbV.E4B was abundantly expressed in the cortex (Figures S9A and S9B), sprouting into the ipsilateral spinal cord was increased at the cervical level in the sensorimotor cortices of UbV.E4B-treated rats, and more axons extended into the contralateral denervated spinal cord through the midline (Figures 6C and 6D). In other words, sprouting of CST axons into the ipsilateral spinal cord was markedly increased in UbV.E4B-treated rats, whereas limited sprouting was observed in the controls (Figures 6C and 6D). By analyzing the video recordings of the behavioral performances of rats in a double-blinded manner, we found that rats treated with UbV.E4B exhibited significant recovery of forelimb function in both tasks (Figures 6E and 6G), indicating that CST axon regrowth contributed to the improvement of fine forelimb motor functions after stroke. More specifically, UbV.E4B treatment significantly increased the overall success rate in the pellet retrieval test (Figure 6E). According to scores assigned based on the Eshkol-Wachman Movement Notation (EWMN) system, improvements in the grasping component of the pellet retrieval test, but not the reaching or retrieval component, mainly contributed to the observed functional recovery of the forelimb (Figure 6F). Moreover, although the reaching trajectories of the rats were highly variable after stroke (Figure S10A), a significant difference in trajectory variability was not observed compared with that in the ubiquitin-treated group (Figure S10C). The endpoint distributions also showed no obvious differences among groups (Figures S10B and S10D), further verifying that improvements in grasping, but not improvements in reaching, primarily contributed to the observed functional recovery in the pellet retrieval test.

### Ablation of sprouting CST axons abolishes the recovery of skilled locomotor ability

**Figure 7:**
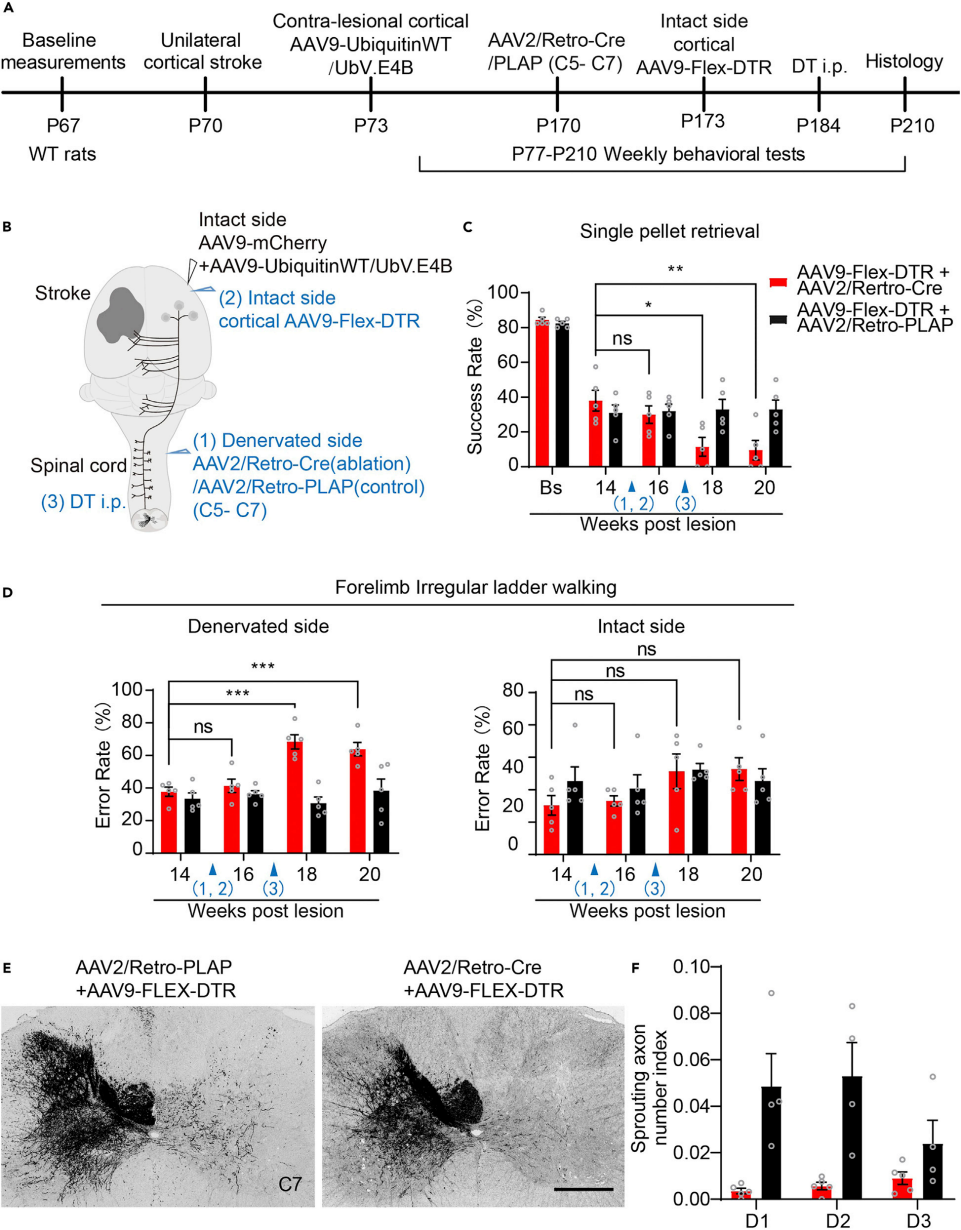
Ablation of sprouted corticospinal neurons axons abolishes the recovery of skilled locomotor ability (A and B) Time course (A) and schematic (B) of the experimental procedure. Rats that received AAV9-UbV.E4B treatment after stroke were intraspinally injected (C5–C7) with AAV2/Retro-Cre (ablation) or AAV2/Retro-PLAP (control) on the denervated side at P170 (1) and cortically injected with AAV9-Flex-DTR (intact side) at P173 (2). At P184, DT was administered (i.p.) (3). (C and D) The functions of the forelimb on the intact and denervated sides in the single-pellet retrieval task (C) and irregular horizontal ladder tasks were analyzed (D). ∗p < 0.05, ∗∗p < 0.01, and ns, not significant (Student’s *t* test; n = 5 animals per group). (E) Representative images of transverse C7 spinal cord sections immunostained for RFP to label the CST axons originating from the intact side in animals that received an intraspinal injection of AAV2/Retro-PLAP (control) or AAV2/Retro-Cre (ablation). The scale bar represents 500 μm. (F) Quantification of midline-crossing axons in the cervical spinal cord (C7) of animals that received an intraspinal injection of AAV2/Retro-PLAP (control) or AAV2/Retro-Cre (ablation). ∗∗p < 0.01; ns, not statistically significant. Student’s *t* test; n = 5 in the AAV2/Retro-Cre (ablation)-injected group; n = 4 in the AAV2/Retro-PLAP (control)-injected group. Three serial sections of the C7 spinal cord were quantified for each rat. The data are presented as the means ± SEM in (C), (D) and (F).

We selectively ablated sprouting axons in the cervical spinal cord by administering an intraperitoneal (i.p.) injection of diphtheria toxoid (DT) after a unilateral injection of AAV2/Retro-Cre into the denervated side of the cervical spinal cord (C5–C7) and an injection of AAV9-Flex-DTR (ablation) or AAV9-Flex-PLAP (control) into the intact side of the cortex in a set of rats expressing UbV.E4B, as described above, to further verify the contribution of CST sprouting in the cervical spinal cord to functional recovery (Figures 7A and 7B). We verified that the behavioral performance of the rats was not altered by these intraspinal and cortical injections (Figures 7C and 7D). However, 2 weeks after DT administration, the improved performance of rats expressing UbV.E4B on the single-pellet retrieval task and irregular ladder walking of the denervated forelimb was remarkably abolished. On the other hand, a significant difference in the performance of the intact forelimb on irregular ladder walking was not observed (Figures 7C and 7D). Consistently, ablation of CST axons was observed in the cervical spinal cord on the denervated side (Figures 7E and 7F). Thus, our results suggested that axonal sprouting in the spinal cord is required for the recovery of skilled motor ability after unilateral photothrombotic stroke.

## Discussion

Using *in vivo* CRISPR screening and gene manipulation strategies, we found that UBE4B simultaneously modulates two of major pathways that control optic nerve and CST axon regrowth, i.e., the p53 and KLHL22/mTOR pathways. We developed an AAV-based method for overexpressing UbV.E4B that mimics axonal regrowth triggered by UBE4B deletion to translate this finding into a potential translational strategy for restoring CST-dependent functions after cortical stroke in clinical applications.

Surprisingly, we found that among molecules involved in ubiquitin pathways, UBE4B, but not MDM4, played a major role in regulating CNS axon regrowth. A previous study reported that conditional deletion of MDM4 promotes axon regeneration.^11^ In our study, we observed little regrowth of optic nerve axons in mice with MDM4 deletion, but UBE4B deletion induced much more robust axon regeneration after ONC injury. Although MDM4 might also play a role in regulating axonal regrowth via the p53 pathway, UBE4B activates the KLHL22/mTOR pathway in addition to the p53 pathway. This finding explains why UBE4B deletion exerted a superior effect on promoting axonal regrowth and emphasizes the importance of considering multiple pathways when designing interventions for promoting functional recovery through axonal regrowth.

Previously, several strategies, such as activation of mTOR and/or overexpression of p53, have been proposed to promote CNS axon regrowth.^8^^,^^38^^,^^39^^,^^40^ The mTOR signaling pathway has been suggested to play an important role in synaptogenesis and differentiation, particularly axon regeneration and neuronal survival after CNS injury.^38^^,^^41^ P53, a well-known tumor suppressor, is a multifunctional sensor of a number of cellular signals and pathways essential for angiogenesis, cell metabolism, DNA damage, cell cycle regulation, apoptosis, and nerve regrowth.^39^^,^^42^^,^^43^ However, the key molecule involved in the simultaneous modulation of these two important pathways was not identified previously. In the present study, UBE4B, which has been proven to promote p53 polyubiquitination and degradation and inhibit p53-dependent transactivation and apoptosis,^19^ played a major role in CNS axon regrowth. Further analysis showed that UBE4B, in addition to p53, activated the mTOR pathway via its substrate KLHL22 to promote axon regrowth, revealing a new mechanism underlying the regulation of CNS axon regrowth.

In a rat cortical stroke model, we showed that overexpression of UbV.E4B in cortical neurons restored CST-dependent function by promoting CST axon regrowth. Although numerous intrinsic mechanisms underlying the regulation of CNS axon regrowth have been explored, few have been studied in translatable settings.^1^^,^^44^ Here, in a translatable setting, we showed that overexpression of UbV.E4B alone was sufficient to trigger robust CST axon regrowth and functional recovery, suggesting that UbV.E4B is a potential treatment for cortical stroke. However, regenerating CST axons might need to be fine-tuned to better perform their functions. Because exercise and electrical stimulation promote the plasticity of CST axons toward the development of functional spinal networks,^36^^,^^44^^,^^45^^,^^46^^,^^47^ the combination of electrical stimulation and UbV.E4B overexpression might further improve functional recovery after cortical stroke. Studies examining this combination strategy and the potential of systemic UbV.E4B delivery as a treatment for cortical stroke are ongoing.

### Limitations of the study

First, downregulation of UBE4B promotes axon regeneration without enhancing the survival rate of RGCs after injury; whether there are new strategies beneficial to not only RGC survival but also axon regeneration is worth exploring. Second, besides mTOR and p53, other druggable targets for UBE4B might need to be explored in future study because there are numerous substrates for UBE4B. Third, AAV gene delivery is featured by high price and technical difficulty to safely deliver genes to the CNS. Last, regenerating CST axons might need to be fine-tuned by combination of electrical stimulation, UbV.E4B overexpression, and so on, to better reinvent functional spinal networks and perform their functions after cortical stroke.

## STAR★Methods

### Key resources table

**Table:** 

REAGENT or RESOURCE	SOURCE	IDENTIFIER
**Antibodies**		

Rabbit anti-UBE4B	Invitrogen	PA5-22023
Rabbit anti-RBPMS	Abcam	Cat# ab194213, RRID:AB_2920590)
Rabbit anti-phosphorylated S6 Ser235/236	Cell Signaling	Cat# 4857, RRID:AB_2181035
Rabbit anti-RFP	Abcam	Cat# ab34771, RRID:AB_777699
Rabbit anti-KLHL22	ProteinTech	Cat# 16214-1-AP, RRID:AB_2131201
Rabbit anti-Tuj1	Cell Signaling	Cat# 5568, RRID:AB_10694505
Rabbit anti-GAPDH	ABclonal	Cat# A19056, RRID:AB_2862549
Mouse anti-HA.11 Epitope Tag	Biolegend	Cat# 901516, RRID:AB_2820200
Mouse anti-TUJ1	BioLegend	Cat# 801213, RRID:AB_2728521
Mouse anti-p53	Cell Signaling	Cat# 2524, RRID:AB_331743
Mouse anti-mTOR	Cell Signaling	Cat# 9964, RRID:AB_10696892
Mouse anti-PKCγ	Santa Cruz	Cat# sc-166385, RRID:AB_2018059
Mouse anti-ATF3	Santa Cruz	sc-518032
Mouse anti-p-c-Jun	Santa Cruz	Cat# sc-822, RRID:AB_627262
Goat anti-rabbit IgG (H+L) secondary antibody, HRP	Invitrogen	31460
Goat anti-mouse IgG (H+L) secondary antibody, HRP	Invitrogen	31430
Donkey anti-rabbit, Alexa Fluor 488	Abcam	Cat# ab150073, RRID:AB_2636877
Donkey anti-mouse, Alexa Fluor 488	Abcam	Cat# ab7057, RRID:AB_955251
Donkey anti-mouse, Alexa Fluor 647	Abcam	Cat# ab150111, RRID:AB_2890625
Donkey anti-rabbit, Alexa Fluor 647	Bioss	Cat# bs-0295D-A647, RRID:AB_10891079
Donkey anti-rabbit, Alexa Fluor 555	Invitrogen	A-31572

**Recombinant DNA**		

pAAV-CAG-Cre-WPRE-hGH	ZJU Viral Core	N/A
pAAV-CMV-PLAP-hGH	ZJU Viral Core	N/A
pAAV-CAG-CNTF-WPRE	Vigene Biotechnologies	N/A
pAAV-U6-gRNA (for UBE4B; 5 gRNA pools)-CAGminin-GFP	Vigene Biotechnologies	Table S2
pAAV-U6-gRNA (for Pirh2; 5 gRNA pools)-CAGminin-GFP	Vigene Biotechnologies	Table S2
pAAV-U6-gRNA (for MDM4; 5 gRNA pools)-CAGminin-GFP	Vigene Biotechnologies	Table S2
pAAV-U6-gRNA (for HAUSP; 5 gRNA pools)-CAGminin-GFP	Vigene Biotechnologies	Table S2
pAAV-U6-gRNA (for Cop1; 5 gRNA pools)-CAGminin-GFP	Vigene Biotechnologies	Table S2
pAAV-U6-gRNA (for p53; 2 gRNA pools)-CAGminin-GFP	ZJU Viral Core	Table S2
pAAV-U6-gRNA (for KLHL22; 2 gRNA pools)-CAGminin-GFP	ZJU Viral Core	Table S2
pAAV-CAG-HA-UbiquitinWT-WPRE	ZJU Viral Core	Table S3
pAAV-CAG-HA-UbV.E4B-WPRE	ZJU Viral Core	Table S3

**Software and algorithms**		

ImageJ	NIH	https://imagej.nih.gov/ij/
Prism	GraphPad	https://www.graphpad.com/
Adobe Illustrator CC 2018	Adobe Systems	https://www.adobe.com/products/illustrator.html
calClosure algorithm	This paper; Closure Data	https://doi.org/10.5281/zenodo.7375563
calHausdorff algorithm	This paper; Hausdorff Data	https://doi.org/10.5281/zenodo.7375746

### Resource availability

#### Lead contact

Further information and requests for resources and reagents should be directed to and will be fulfilled by the lead contact, Xuhua Wang (xhw@zju.edu.cn).

#### Materials availability

This study did not generate new unique reagents.

### Experimental model and subject details

#### Mouse lines

Original mouse lines, including the flox-UBE4B-flox and Rosa26-LSL-Cas9 knock-in lines, were gifts from the Zhiping Wang laboratory, Medical School of Zhejiang University. Rosa26-LSL-Cas9 knock-in mice were crossed with flox-UBE4B-flox mice to generate flox-UBE4B-flox and flox-STOP-flox-Cas9-GFP homozygotes (UBE4B^f/f^-LSL-Cas9, harvested from F2). Through the delivery of CRE and sgRNAs via AAV vectors, UBE4B and another target gene were knocked out simultaneously in the retina. C57BL/6 mice (both gender, 4-week-old) were purchased from Shanghai SLAC Laboratory Animal Co. Sprague‒Dawley rats (200–250 g, female) were purchased from the Experimental Animal Center of Zhejiang Academy of Medical Science, Hangzhou, China.

#### Mouse husbandry

The mice were bred and maintained at the Experimental Animal Center of Zhejiang University. All experiments were approved by the Zhejiang University School of Medicine Animal Experimentation Committee (approval IDs: ZJU20210110 and ZJU20210153). The mice were provided *ad libitum* access to food and water and housed in cages with positive-pressure filtered air. The bedding changed frequently. The mice were not permitted to breed before or during their inclusion in the *in vivo* experiments. For surgical procedures, mice were anesthetized with avertin. Animals of both sexes were used.

### Method details

#### Production of AAVs

The AAV2-U6-sgPirh2, AAV2-U6-sgCop1, AAV2-U6-sgHAUSP, AAV2-U6-sgMDM4, AAV2-U6-sgUBE4B, AAV2-U6-sgPTEN, AAV2-CAG-Cre-WPRE, AAV9-hSyn-mCherry, AAV9-CAG-PLAP, and AAV9-Flex-DTR vectors were purchased from Vigene Biosciences. The AAV2-UbV.E4B and AAV2-UbiquitinWT vectors were generated by the Viral Core of Zhejiang University. The sequences of the sgRNAs, UbV.E4B and UbiquitinWT are listed in Tables S2 and S3. All AAV vectors were packaged into AAV2, AAV9, or AAV2/Retro by the Viral Core of Zhejiang University and titrated to 1X10^13^ genome copies per milliliter for injection.

#### Intravitreal injection and ONC

For intravitreal injection, animals were anesthetized with avertin, and then the edge of the eyelid was clamped with a small artery clamp to expose the conjunctiva. AAV (1–3 μL) was injected intravitreally on postnatal day (P)28, and Alexa Fluor-conjugated CTB (CTB-555, 1 mg/mL; 1–2 μL, Invitrogen) was injected intravitreally at P54. The agents were injected with a fine glass pipette attached to the Hamilton syringe using plastic tubing. The CTB-555 injection was performed 2–3 days before euthanasia to anterogradely trace regenerating RGC axons. Mice with obvious eye inflammation or shrinkage were sacrificed and excluded from further experiments. Two weeks after the virus injection, intraorbital ONC was performed. After the mice were anesthetized and an incision was made in the conjunctiva, the optic nerve was crushed using a pair of forceps with a 0.1 mm-wide tip for 5 s 1–2 mm behind the optic disk.

#### Unilateral photothrombotic stroke

Animals were fixed in a stereotactic frame, and the skull was exposed. A cold light source (WeiHaiLiXin, LX-D40, 40 W, 9000 mW/cm^2^) was positioned over an opaque template with an opening (a 10 mm × 5 mm rectangle for rats or a circle with a diameter of 2.5 mm for mice) to target the sensorimotor cortex corresponding to the preferred paw. For rats, Rose Bengal (20 mg/kg body weight, 20 mg/mL Rose Bengal in saline) was injected via the tail vein, and after 2 min, the brain was illuminated through the skull for 15 min. For mice, Rose Bengal (10 mg/kg body weight, 5 mg/mL Rose Bengal in saline) was injected via the tail vein 10 min before the brain was illuminated through the intact skull for 15 min.

#### Virus injection

Neonatal Ube4B^f/f^ mice were cryo-anesthetized for 30 s and 3 μL of either AAV9-Cre or AAV9-PLAP were injected into the right sensorimotor cortex using a 10-μL Hamilton microsyringe with a pulled-glass micropipette (68606, RWD, China). After the injection, the mice were placed on a heating pad and returned to their mothers after they regained normal color and activity. Six weeks later, unilateral photothrombotic stroke was induced in the left sensorimotor cortex. We injected 4 μL of AAV9-mCherry into the sensorimotor cortex 6 weeks after stroke at a rate of 80 nL min^−1^ (300 nL per site, twelve sites) to anterogradely label the CST. Mice were maintained for an additional 2 weeks before being euthanized. All AAV vectors, including AAV9-mCherry and AAV9-Cre/PLAP, were generated at the Viral Core of Zhejiang University, and their titers were adjusted to 1X10^13^ copies per mL for injection. The rats were injected with 3 μL of AAV9-UbiquitinWT/UbV.E4B and AAV9-mCherry into the contralesional cortex at a rate of 150 nL min^−1^ (150 nL per site, eighteen sites) three days after stroke. The rats were placed on soft bedding on a heated blanket maintained at 37°C until they were fully awake.

#### Selective ablation of CSNs with axons sprouting into the denervated side of the spinal cord

Rats underwent unilateral photothrombotic stroke at P70 and were treated with AAV9-UbiquitinWT/UbV.E4B at P73. Fourteen weeks after injury, laminectomy was performed at the cervical level. AAV2/Retro-Cre (ablation) or AAV2/Retro-PLAP (control) was stereotaxically injected into the denervated side of the cervical (C5–C7) spinal cords of AAV9-UbV.E4B-treated rats using the protocol established by previous literatures.^1^^,^^48^ AAV9-Flex-DTR was then injected into the unlesioned sensorimotor cortex 3 days after the AAV2/Retro-Cre/PLAP injection. After 2 weeks, the animals were subjected to the irregularly spaced horizontal ladder walking task and/or single-pellet retrieval task to reassess skilled limb movement. Diphtheria toxin was then administered (100 mg/kg, i.p.). The animals were subjected to the horizontal ladder walking task and/or single-pellet retrieval task again at 2 and 4 weeks after diphtheria toxin administration.

#### Tissue preparation

Anesthetized animals were transcardially perfused with 4% paraformaldehyde (PFA). Dissected tissues were postfixed with 4% PFA overnight and then cryoprotected in 15% and 30% sucrose before being embedded and snap-frozen in OCT. Typically, the optic nerve samples were cut into 10 μm-thick sections, retinal tissues were cut into 20 μm-thick sections, and spinal cord tissues were cut into 25 μm-thick sections. The sections were adhered to room temperature charged microscope slides, dried, and frozen until further processing. Sections were then either washed and mounted with antifade reagent for imaging (for example, CTB labeling of optic nerves) or further processed for immunohistochemistry. Some retinas were dissected *in toto* after postfixation with PFA, washed with PBS, immunostained, cut radially with scissors to flatten the tissue, and then mounted for imaging.

#### Staining

Tuj1 staining of whole mounts was performed to determine the number of surviving RGCs two weeks after ONC. The retinas were washed with 1X PBS three times in a 96-well plate and then blocked for one hour in PBS containing 5% donkey serum and 0.3% Triton X-100. After incubation with a Tuj1 primary antibody diluted in PBS supplemented with 3% donkey serum and 0.3% Triton X-100 for 0.5–2 days at 4°C, the retinas were washed three times with PBS and incubated with the secondary antibody for 1–2 h at room temperature. After the tissues were washed with PBS, the retinas were mounted on glass slides, and images were captured under a wide-field fluorescence microscope (VS120, Olympus, Japan). Twelve images of different quarters covering the peripheral and central regions of each retina were captured. An individual who was blinded to the groups counted the number of Tuj1^+^ RGCs.

Immunohistochemical staining was performed by blocking the sections with 5% normal donkey serum and 0.5% Triton X-100 in PBS and incubating them with primary antibodies overnight at 4°C in blocking solution. After three washes with PBS, sections were incubated with appropriate secondary antibodies conjugated to fluorescent dyes at room temperature.

For TTC staining, rat brains were harvested 1 day after stroke. The brain was dissected and removed promptly and cut into 1.5 mm cortical slices. Sections were immediately stained in a prewarmed 2% 2,3,5-triphenyltetrazolium chloride (TTC) (T8877, Sigma‒Aldrich) solution in saline, pH 7.4, for 10 min at 37°C. Sections were then washed, fixed with 4% PFA overnight at 4°C, and imaged. The area devoid of red staining was the infarct area.

#### Western blot analysis

The mice were lightly anesthetized with isoflurane and then decapitated. The brain tissues were dissected and then homogenized in RIPA buffer containing a protease inhibitor cocktail. After centrifugation, the protein concentration in the supernatant was quantified using the BCA assay. Equal amounts of total protein were electrophoresed on SDS-polyacrylamide gels. The separated proteins were transferred onto polyvinylidene fluoride membranes at 4°C. The membranes were blocked for 2 h with 5% milk in Tris-buffered saline with Tween-20 (TBST) at room temperature and incubated with the primary antibody at 4°C overnight. After washing, the membranes were incubated with the appropriate HRP-coupled secondary antibody for 1 h. Then, the protein bands were detected using an ECL kit according to the manufacturer’s instructions. We also probed the membranes with an antibody against GAPDH (1:1000, ABclonal A19056) to verify equal loading. The density of the immunoblot bands was measured with ImageJ software (NIH, Bethesda, MD).

#### Microscopy

For some retinal sections and whole-mount retinas, individual fluorescence images were acquired using a wide-field fluorescence microscope (VS120, Olympus, Japan). For nerve and spinal cord sections, images were captured using a confocal laser scanning microscope (A1R, Nikon, Japan) with automated tiling, and Z stacks were projected onto a single plane. The brightness and contrast of the images were adjusted, and pseudocolors were applied for presentation. When images were used for quantification, the imaging and processing parameters were kept constant.

#### Behavioral tests

Thirty adult female Sprague–Dawley rats (200–250 g, 3–4 months of age) were used for histological and behavioral studies. The rats were trained in the single-pellet grasping task and irregular ladder walking task. After 3–4 weeks of training, the baseline performance of each rat was recorded, and only animals that achieved an 80% success rate in the single-pellet grasping task and a 25% error rate in the irregular ladder walking task were included in further experiments. Then, video recordings of the behavioral performances of rats were analyzed in a double-blinded manner.

#### Single-pellet grasping task

Each rat was placed in a chamber (45 cm × 13 cm x 40 cm) and allowed to reach for and grasp a pellet (dustless precision pellet, 45 mg, Bioserv) on a shelf through a wide silt in the front of the chamber. The rats were food-restricted to maintain a weight greater than 90% of the free feeding weight throughout the training session. During the test session, 20 pellets were provided within 10 min. The success rate was calculated as the total score/20, where the score was determined according to the criteria listed below. A score of 1 was assigned if the rat directly retrieved the pellet and brought it to its mouth. A score of 0.5 was assigned if the rat successfully grasped the pellet but dropped the pellet inside the box. A score of 0 was assigned if the rat missed the pellet or knocked the pellet off the shelf. All test sessions were videotaped (60 fps) and analyzed further. For a detailed analysis of motor components, scoring based on the EWMN system was performed as described in the original study.^49^

#### Irregular ladder walking task

Rats walked through a ladder, the beams of which were unequally spaced from 1 cm to 5 cm. In each test session, the rats were allowed to walk through the ladder 3 times and videotaped (60 fps) for scoring. The error rate was calculated as the number of error steps/total number of steps. Two types of error steps occurred: 1) Miss: when crossing the ladder, the forelimb either completely missed the rung, or the rung was contacted by the wrist instead of the paw and 2) Slip: when crossing the ladder, the rat placed a few digits instead of the paw on the rungs, causing a subsequent slip. A correct step was defined as precise placement of the center of the palm on the rung and closing of the digits.

### Quantification and statistical analysis

#### Quantification of axon regrowth

First, the experimenters were blinded to the conditions when performing the measurements. The optic nerve was dissected carefully, and longitudinal sections of optic nerves were cryosectioned (section thickness: 10 μm) to quantify the regeneration of axons traced with fluorescent CTB after ONC. Serial optic nerve sections were imaged under a confocal microscope (A1R, Nikon, Japan; 20X objective) using the CTB channel. Images of the nerve sections were exported and viewed in ImageJ software. CTB^+^ axons along the optic nerve were counted at multiple distances (250 μm, 500 μm, 1000 μm, 1500 μm, 2000 μm, etc.) in the anterograde direction from the crush site. At least three images per optic nerve were analyzed. The counts were transformed into axonal density and then multiplied by the approximate cross-sectional area of the nerve (estimated diameter = 250 μm) to estimate the total number of axons on each respective nerve. The following formula was used to calculate the estimated number of regenerated axons at different distances from the crush site on each optic nerve: ad = πr2 ∗ (axon numbers/μm)/[10 μm∗(nerve width at the counting site)]. The average estimate for each optic nerve was recorded as a single value for statistical analysis.

A horizontal line was first drawn through the central canal and across the lateral rim of the gray matter, and fibers crossing the spinal cord midline were counted at 20x magnification to quantify the number of sprouting axons. Four vertical lines (main tract, D1, D2 and D3) were drawn from the central canal to the lateral rim to divide the horizontal line into three equal parts. Although the main tract was used to identify midline crossing fibers, D1, D2 and D3 were used to identify sprouting fibers at different distances from the midline. Only fibers that crossed the four lines were counted in each section. The number of sprouting fibers normalized to the number of CST fibers at the medulla level is presented. Images were imported into and analyzed using Python to erode all CST axons to a single pixel width and determine the distribution of sprouted CST axons. The total number of pixels in a particular area thus corresponded to the overall density of the CST label in a specific area of the section. The pixelated data were further processed using Python to generate heatmaps, with red representing the highest axonal density, blue representing the lowest axonal density and white representing the background of the images. For correlation analyses between regenerated CST axons and the success rate in the single-pellet retrieval test, the average number of axons in each animal was plotted.

#### Quantification of RGC bodies

For quantification of RGCs, eyeballs were cryosectioned at a thickness of 20 μm. The sections were stained for RBPMS (a selective marker of ganglion cells in the mammalian retina) to identify RGCs. Fluorescence images were acquired with a 10x objective using a fluorescence microscope (VS120, Olympus, Japan). At least three sections near the maximum diameter of the eyeball per retina were quantified. In intact control retinas, 300 to 500 RBPMS^+^ cells were generally counted per section. The cell count was normalized to the length of the ganglion cell layer (measured using OlyVIA software for every section), and the average value for each retina was recorded for the subsequent statistical analysis. For quantification of RGCs in whole-mount retinas, fixed whole-mount retinas were first stained for Tuj1, radially cut into a petal shape and viewed under a fluorescence microscope (VS120, Olympus, Japan, 10X objective). Generally, six to eight fields (each 0.4 mm x 0.4 mm) of each retina were used for RGC quantification, and then the values were averaged to obtain one value for each retina for the subsequent statistical analysis.

#### Statistical analysis

All statistical tests were two-tailed, and the sample size (n) was defined as the number of individual eyes, retinas, nerves, mice or rats, as appropriate. GraphPad Prism 8.0 software was used to plot and fit the data. Statistical comparisons were performed using Student’s t test, one-way ANOVA or two-way repeated-measures ANOVA (Bonferroni’s test was used for post hoc analysis). All data are presented as the means ± SEM. In all cases, p < 0.05 was considered statistically significant.

## Data Availability

• The data that support the findings of this study are included in the paper and supplemental information. • All original code has been deposited at Zenodo and is publicly available as of the date of publication (https://doi.org/10.5281/zenodo.7375563; https://doi.org/10.5281/zenodo.7375746). DOIs are listed in the key resources table. • Additional data are available from the corresponding author upon reasonable request.

## Supplemental information

Document S1. Figures S1–S10 and Tables S1–S3

Data S1. Source data for figures, related to all figures
