# Supplementary material for: Downregulation of UBE4B promotes CNS axon regrowth and functional recovery after stroke
Source: iScience. 2022 Dec 26;26(1):105885. doi: 10.1016/j.isci.2022.105885 (PMC9840934; doi:10.1016/j.isci.2022.105885)
Supplement: Document S1. Figures S1–S10 and Tables S1–S3 [file mmc1.pdf]

iScience, Volume *26*

Supplemental information

Downregulation of UBE4B promotes CNS axon regrowth and functional recovery after stroke

Shuang Jin, Xiangfeng Chen, Hanyu Zheng, Wanxiong Cai, Xurong Lin, Xiangxing Kong, Yingchun Ni, Jingjia Ye, Xiaodan Li, Luoan Shen, Binjie Guo, Zeinab Abdelrahman, Songlin Zhou, Susu Mao, Yaxian Wang, Chun Yao, Xiaosong Gu, Bin Yu, Zhiping Wang, and Xuhua Wang

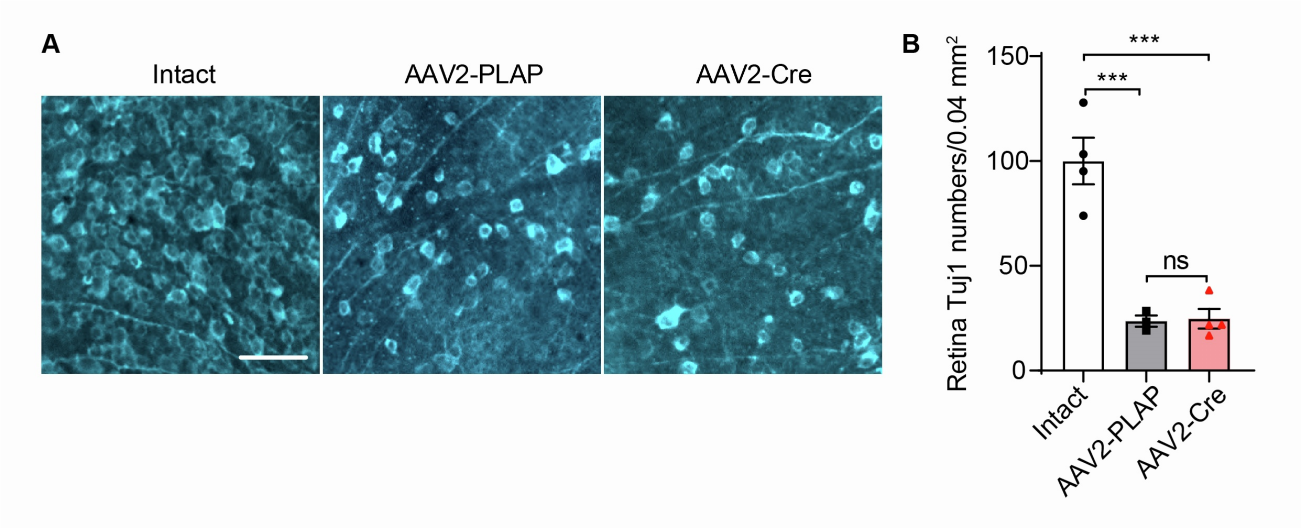

Figure S1. Effects of UBE4B Knockout on RGC Survival after Injury, Related to Figure 2. (A) Representative images of Tuj1-stained whole-mount retinas from intact UBE4B$^{f/f}$ mice injected with AAV2-PLAP and AAV2-Cre at 2 weeks postinjury. The scale bar represents 50 μm. **(B)** Quantification of RGC survival in (A). The data are presented as the means ± SEM (n = 3-4). ***p < 0.001 (ANOVA with Bonferroni's post-test correction).

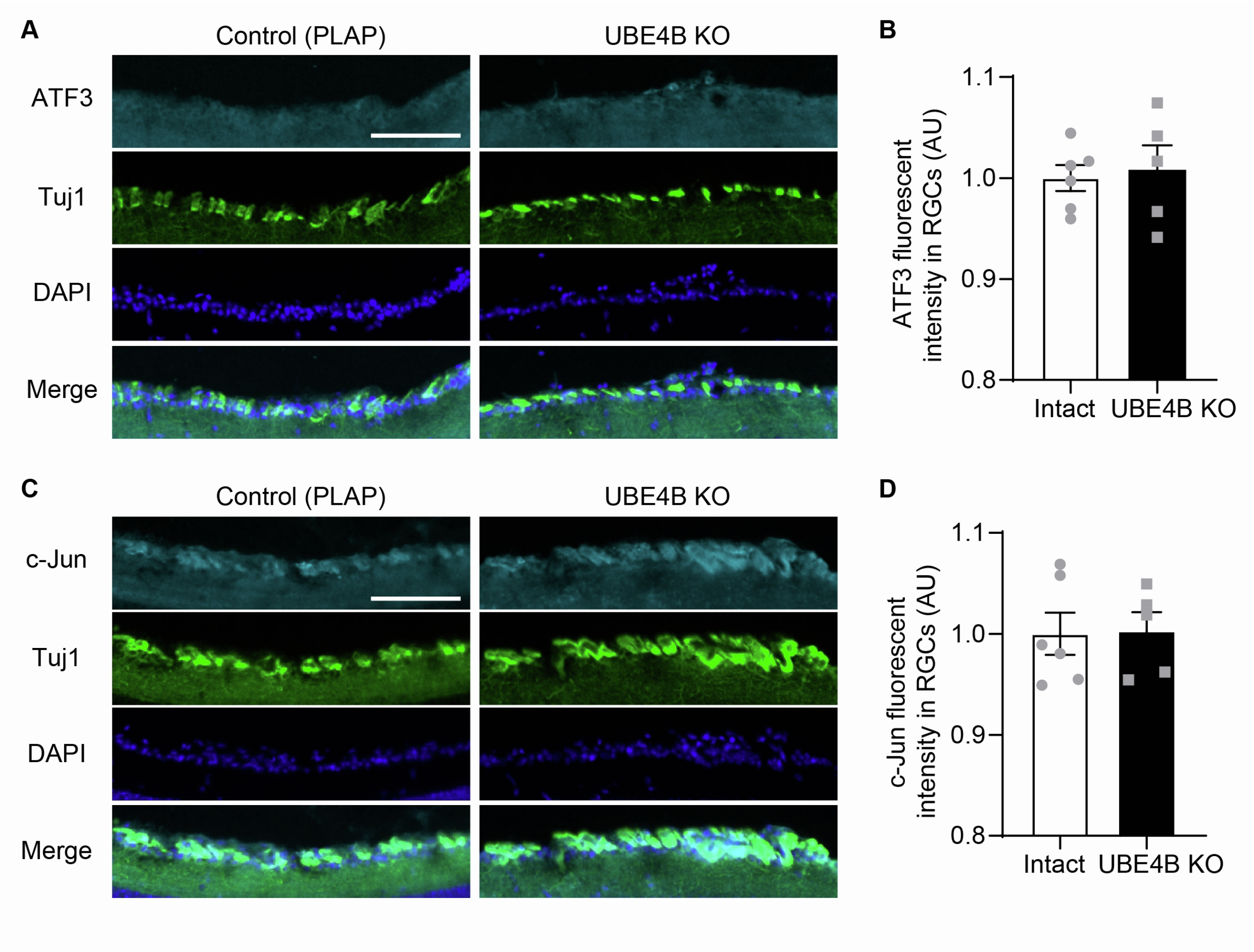

Figure S2. UBE4B Knockout Did Not Activate Stress Response Pathways Prior to Injury, Related to Figure 2. (A) Immunofluorescence staining for ATF3, Tuj1 and DAPI in retinal sections from UBE4B$^{f/f}$ mice injected with AAV2-PLAP or AAV2-Cre. The scale bar represents 100 μm. **(B)** Quantification of the fluorescence intensity of ATF3 in (A). **(C)** Immunofluorescence staining for c-Jun, Tuj1 and DAPI in retinal sections from UBE4B$^{f/f}$ mice injected with AAV2-PLAP or AAV2-Cre. The scale bar represents 100 μm. **(D)** Quantification of the fluorescence intensity of c-Jun in (C). At least three nonconsecutive sections of the ganglion cell layer of retinas from five or six mice per group were used for quantification. The data are presented as the means ± SEM. Student's t test.

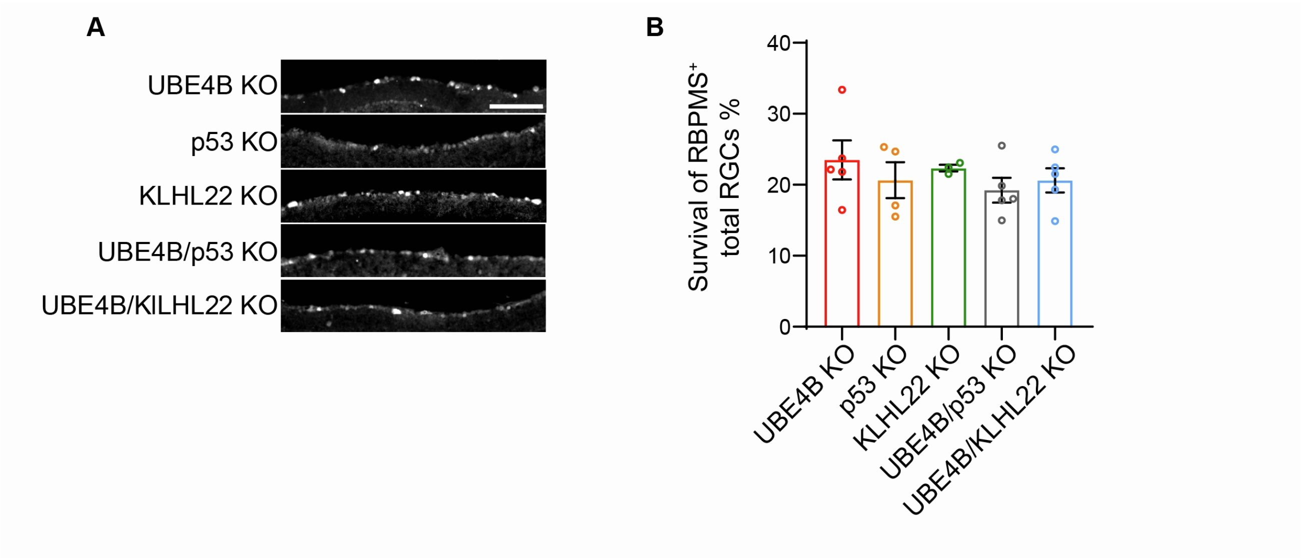

Figure S3. Knockout of p53 or KLHL22 Has No Significant Influence on RGC Survival after Injury, Related to Figure 4. (A) Representative images of RBPMS immunostaining in retinal sections from the different groups. The scale bar represents 100 μm. **(B)** Quantification of the data in (A). The data are presented as the means ± SEM (ANOVA with Bonferroni's post hoc test; n=3-5).

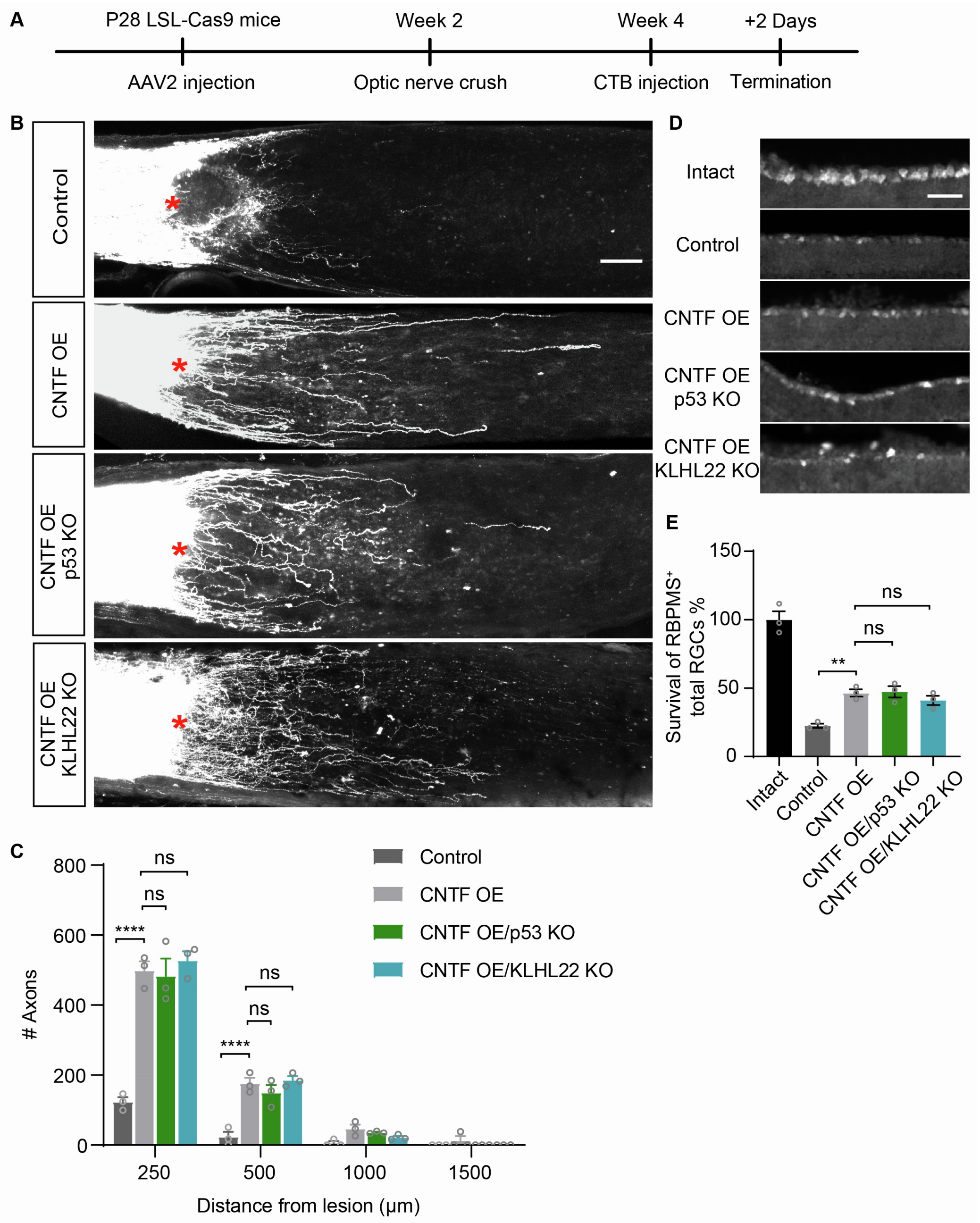

Figure S4. Knockout of p53 or KLHL22 Does Not Affect Regeneration Induced by CNTF Overexpression, Related to Figure 4. (A) Timeline of the experimental procedure used to study optic nerve regeneration. **(B)** Confocal images of optic nerves from the control group, CNTF overexpression group (CNTF OE), CNTF overexpression and p53 knockout group (CNTF OE; p53 KO) or CNTF overexpression and KLHL22 knockout group (CNTF OE; KLHL22 KO) showing CTB-labeled axons around the lesion sites 2 weeks after injury. The crush site is indicated by a red asterisk. The scale bar represents 100 μm. **(C)** Quantification of regenerating axons in (B) at different distances from the injury site (n=3). The data are presented as the means ± SEM (ANOVA with Bonferroni's post hoc test). **(D)** Representative images of RBPMS staining in sections of intact retinas or injured retinas 2 weeks after injury following AAV injection. The scale bar represents 100 μm. **(E)** Quantification of the data in (D). The data are presented as the means ± SEM (n=3-4). ****$p<0.0001$, ns, not significant (ANOVA with Bonferroni's post hoc test).

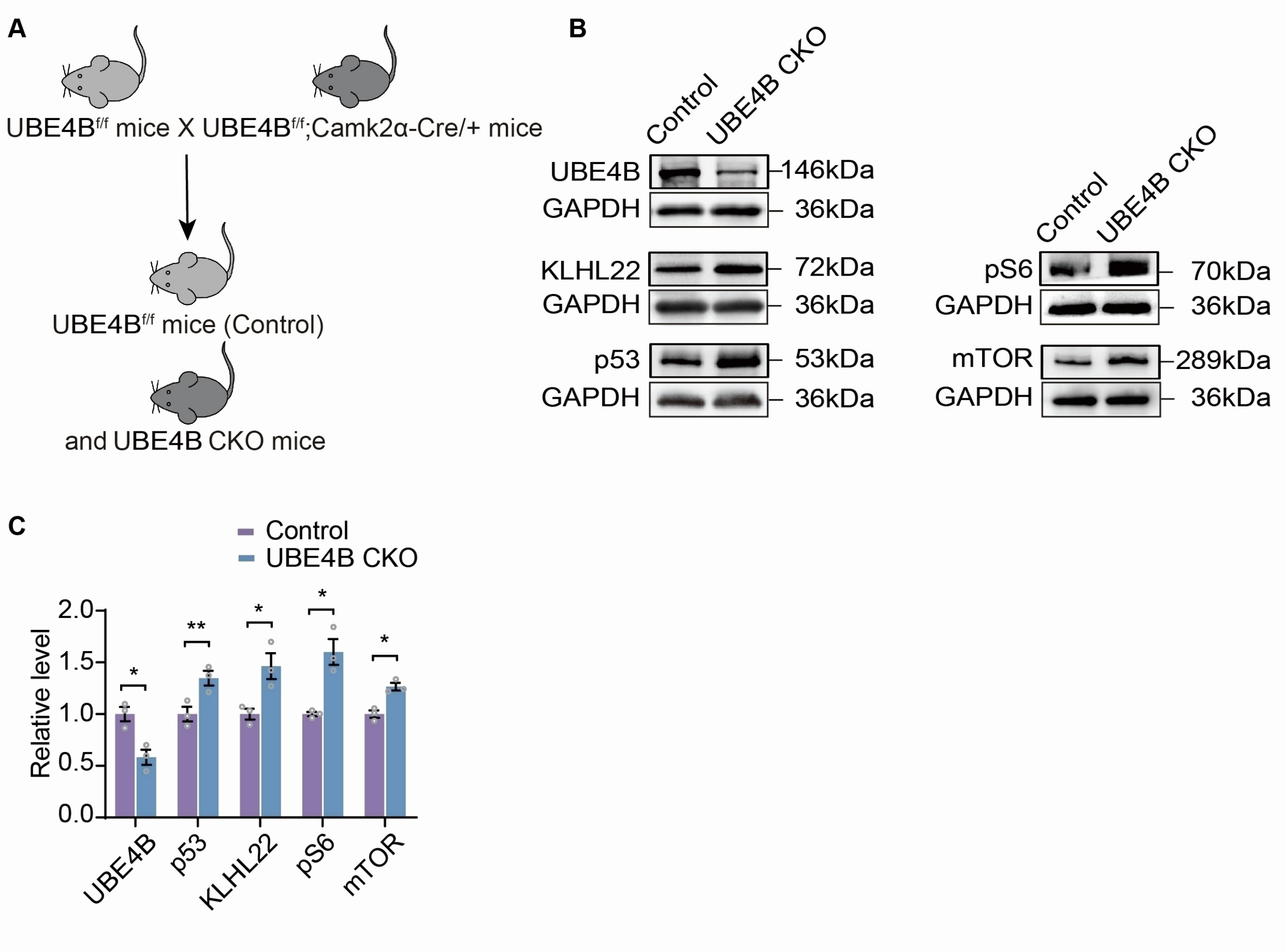

Figure S5. UBE4B Knockout Upregulates p53 and mTOR Expression in the Brain, Related to Figure 4. (A) Diagram showing the strategy used to generate UBE4B$^{f/f}$ mice (control group) and UBE4B conditional knockout mice. **(B)** Representative western blots showing the expression of UBE4B, p53, KLHL22, pS6 and mTOR in the brains of control mice and UBE4B conditional knockout mice. **(C)** Expression of UBE4B, p53, KLHL22, pS6 and mTOR in the brains of UBE4B$^{f/f}$ mice (the control group) and UBE4B conditional knockout mice. The data are presented as the means ± SEM. *$p < 0.05$ and **$p < 0.01$ (Student's t test; n = 3 mice per group).

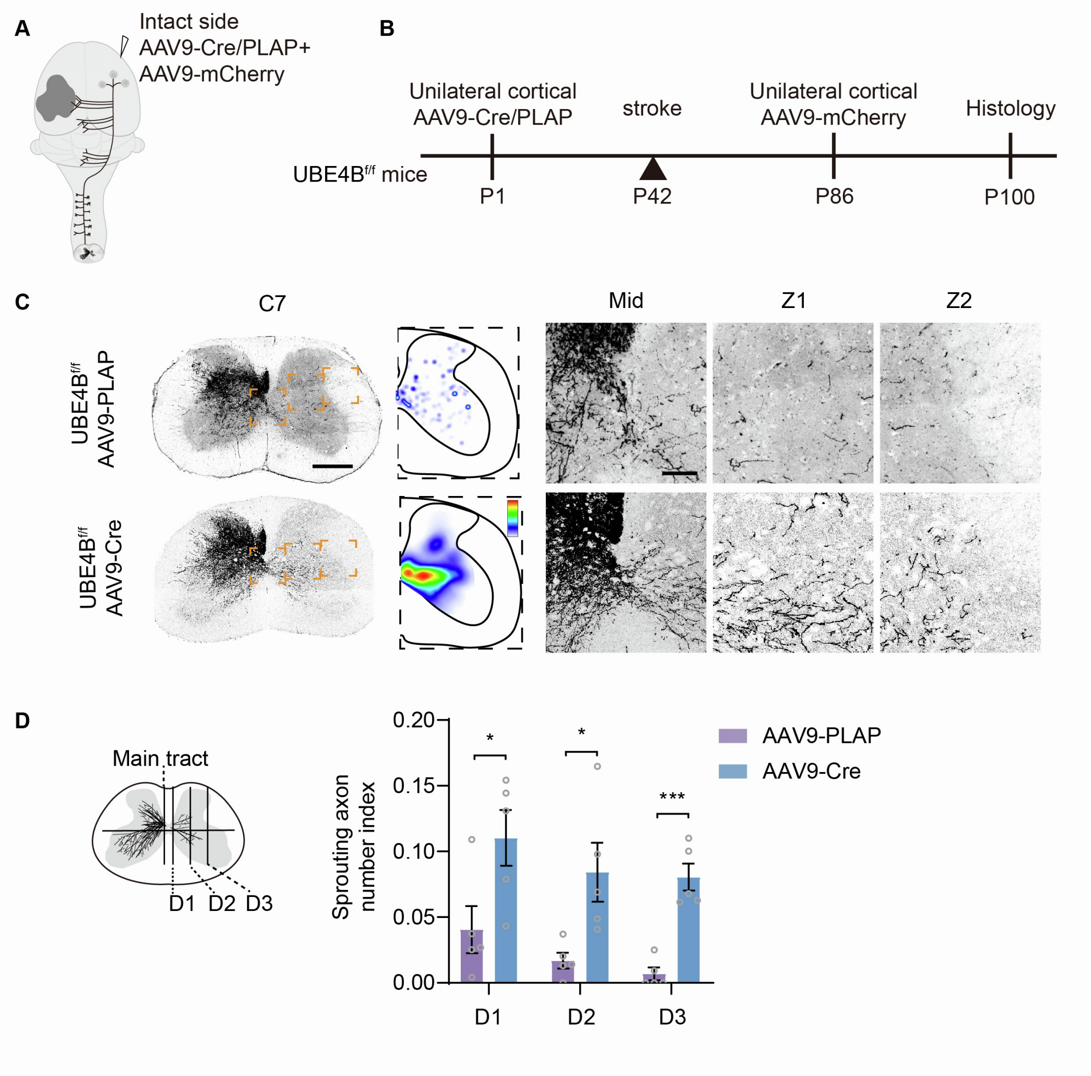

Figure S6. UBE4B Knockout Promotes CST Axon Sprouting in the Spinal Cord after Unilateral Cortical Stroke, Related to Figure 4. (A) Schematic of the experimental procedure. **(B)** Timeline of the experimental procedure. AAV9-PLAP or AAV9-Cre and AAV9-mCherry were injected into the cortices (intact side) of P1 UBE4B$^{f/f}$ mice 6 weeks after unilateral photothrombotic stroke. Collateral sprouting of corticospinal axons occurred in the spinal cord after injury in the control (AAV9-PLAP) and experimental (AAV9-Cre) groups. **(C)** Representative images of transverse sections of the cervical (C7) spinal cord immunostained for RFP to label the CST axons originating from the intact side in animals that received a cortical injection of AAV9-PLAP (control) or AAV9-Cre (UBE4B knockout) (left panels). The distribution of axonal sprouting into the denervated side was visualized in heatmaps (middle panels); red represents the highest numbers of axon pixels, blue represents the lowest numbers of axon pixels, and white represents the background. Right panels in (C): detailed views of the boxed areas in the left panels showing RFP-labeled CST terminal sprouting in denervated areas in the Mid, Z1 or Z2 regions of the cervical spinal cord in control and UBE4B knockout mice. The scale bars represent 500 μm (left panels) and 100 μm (right panels). **(D)** Quantification of midline-crossing axons in the cervical (C7) spinal cord in animals that received an intraspinal injection of AAV9-PLAP (control) or AAV9-Cre (UBE4B knockout). The data are presented as the means ± SEM. *$p < 0.05$ and ***$p < 0.001$ (Student's t test; n = 5 mice per group). At least three serial sections of the C7 spinal cord were quantified for each mouse.

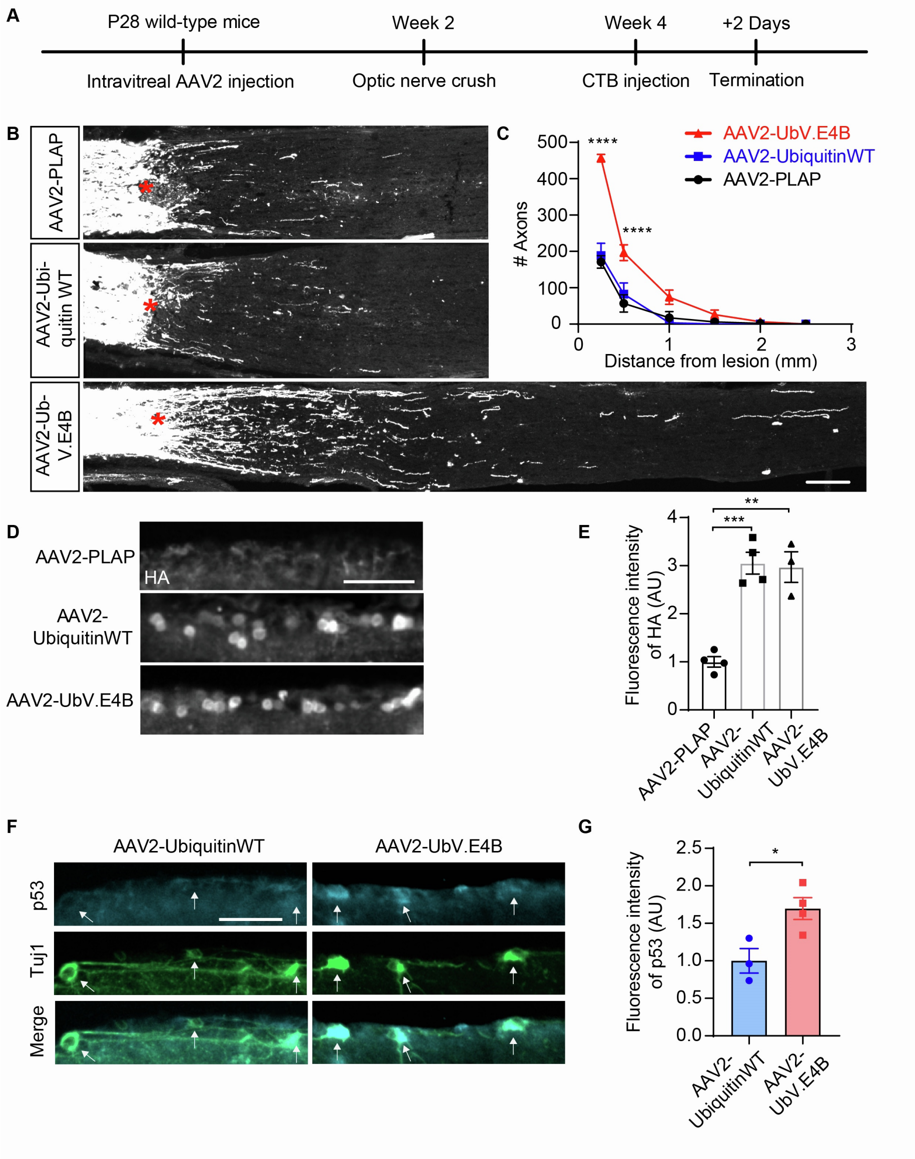

Figure S7. Overexpression of UbV.E4B Promotes Optic Nerve Regeneration, Related to Figure 6. (A) Timeline of the experimental procedure used to study optic nerve regeneration after UbV.E4B treatment. **(B)** Representative images of optic nerve sections from wild-type mice showing CTB-labeled axons following intravitreal injections of AAV2-PLAP, AAV2-UbiquitinWT and AAV2-UbV.E4B 2 weeks after optic nerve injury. The crush site is indicated by a red asterisk. The scale bar represents 100 μm. **(C)** Quantification of regenerating axons in (B). The data are presented as the means ± SEM (n = 3–4). ****p < 0.0001 (ANOVA with Bonferroni's post hoc test, compared to the AAV2-Control group). **(D)** Representative images of retinal sections stained with anti-HA antibodies 2 weeks after injury following the injection of AAV2-Control, AAV2-UbiquitinWT or AAV2-UbV.E4B. The scale bar represents 50 μm. **(E)** Quantification of the fluorescence intensity of HA in (D). **(F)** Immunofluorescence staining for p53 (top panels), Tuj1 (middle panels) and their merge (bottom panels) in sections of retinas from wild-type mice injected with AAV2-PLAP, AAV2-UbiquitinWT or AAV2-UbV.E4B 2 weeks after crush injury. The scale bar represents 100 μm. **(G)** Quantification of the fluorescence intensity of p53 in (F). At least three nonconsecutive sections of the ganglion cell layer of retinas from three or four mice per group were used for quantification. The data are presented as the means ± SEM. *p < 0.05, Student's t test.

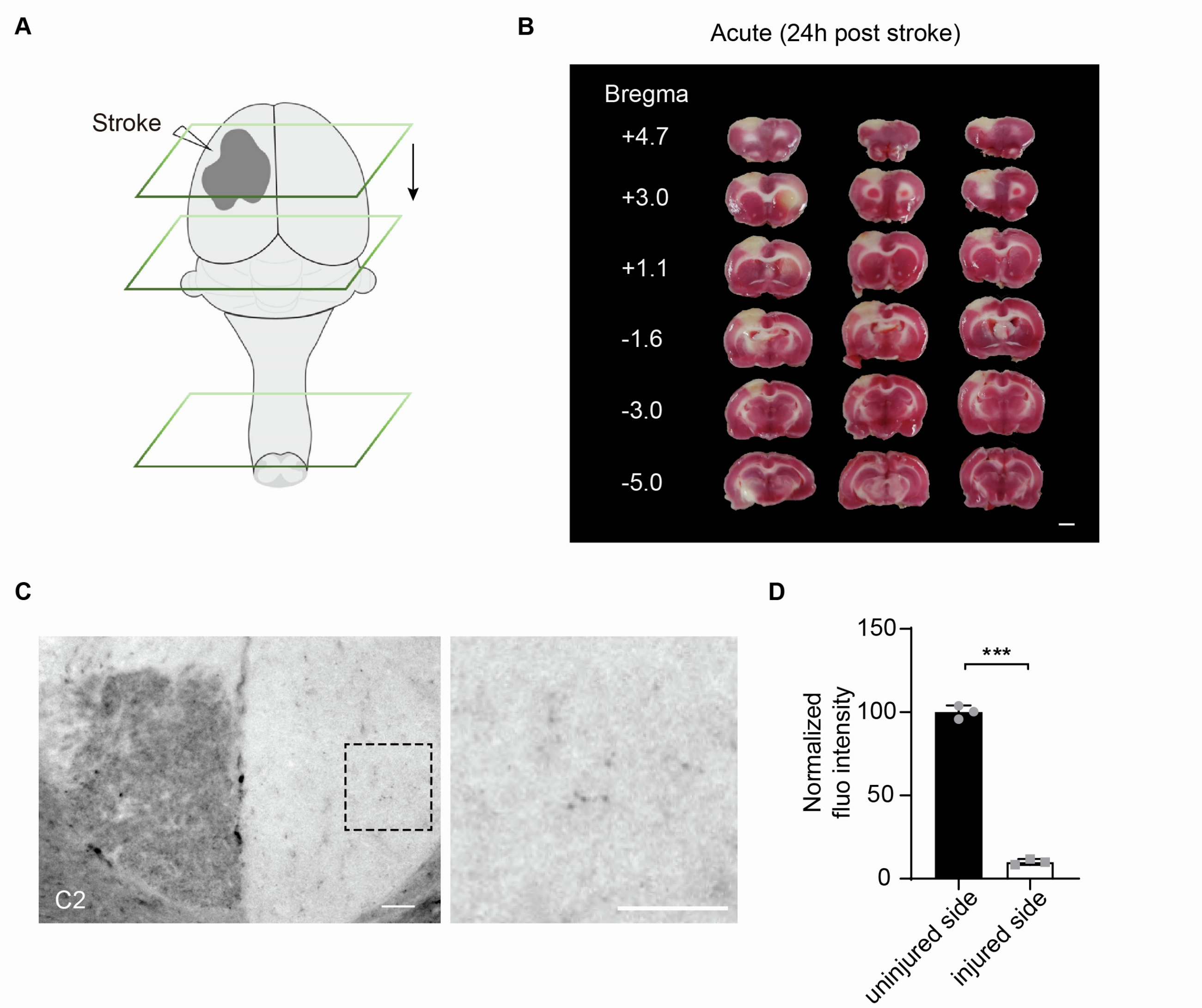

Figure S8. Validation of Unilateral Photothrombotic Stroke, Related to Figure 6. (A) Schematic showing the light-covered areas (sensorimotor cortex) of unilateral photothrombotic stroke. **(B)** Representative images of TTC staining of coronal sections [bregma 4.7, 3.0, 1.1, -1.6, -3.0, and -5.0 (from the upper row to the bottom row)] showing the lesion across the sensorimotor cortex. Scale bar: 2 mm. **(C)** Representative image of the dorsal funiculus at the cervical spinal cord (C2) stained with an antibody against PKCγ. Scale bar: 50 μm. **(D)** Immunofluorescence staining above the threshold was quantified for both the uninjured side and injured side. ***$p < 0.001$, Student's t test, n=3 rats.

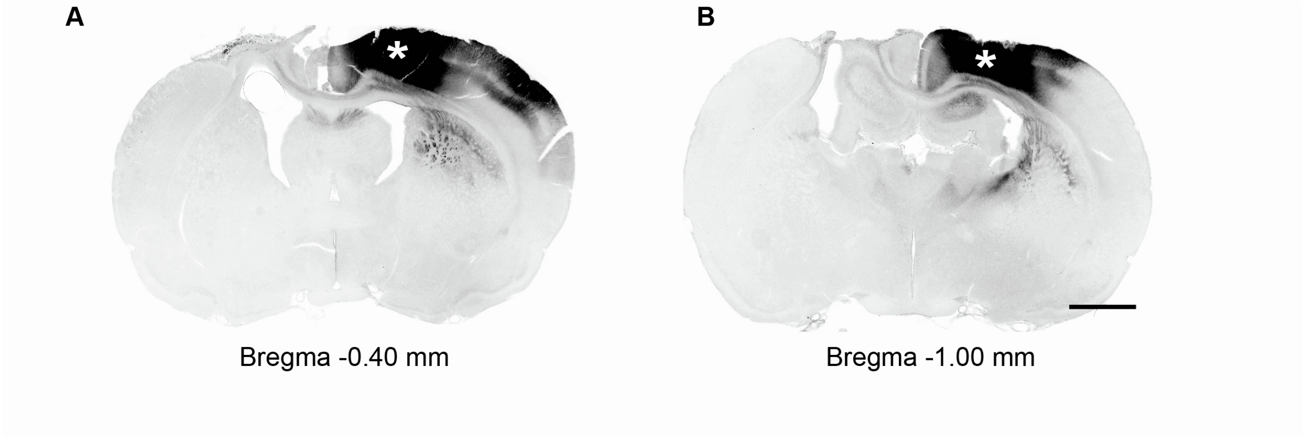

Figure S9. Overexpression of HA-UbV.E4B in the Sensorimotor Cortex, Related to Figure 6. (A and B) HA staining of cortical sections from animals that received the AAV9-UbV.E4B injection at the age of 10 weeks. The asterisk indicates the injection site. Different sections covering the entire sensorimotor cortex are shown. The scale bar represents 1 mm.

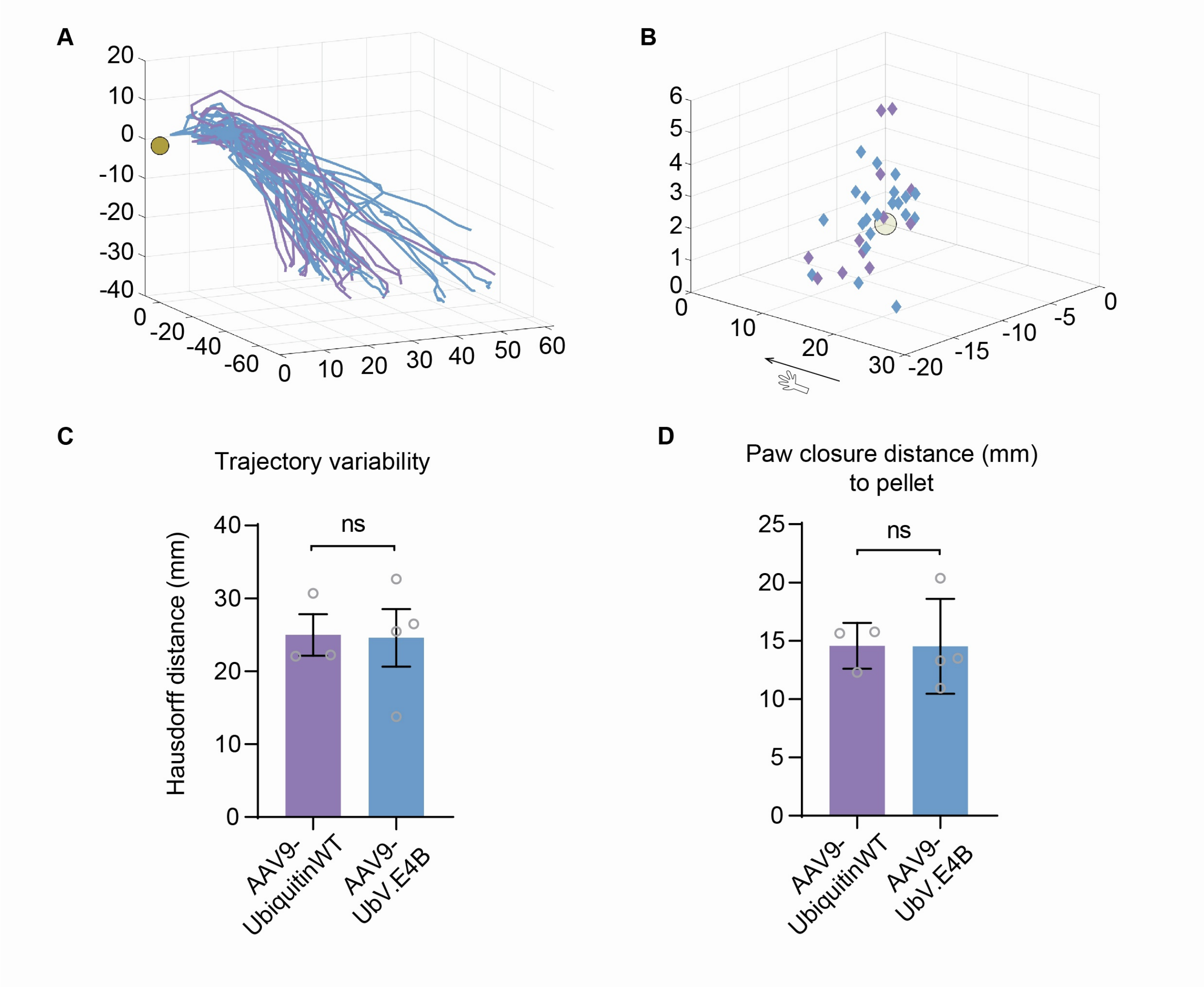

Figure S10. UbV.E4B Treatment Has No Significant Effect on Reaching, Related to Figure 6. (A) Representative three-dimensional reaching trajectories of AAV9-UbiquitinWT- and AAV9-UbV.E4B-injected rats. The brown circles represent the positions of the pellets. **(B)** Representative three-dimensional (x, y, z) paw closure positions relative to the pellet for AAV9-UbiquitinWT- and AAV9-UbV.E4B-injected rats. The paw in the cartoon shows the direction of reaching. The circle represents the pellet, and the center is located at the coordinates (0, 0, 0). **(C)** The Hausdorff distance between multiple trajectories was calculated for AAV9-UbiquitinWT- and AAV9-UbV.E4B-injected rats. **(D)** Distance between the paw closure position and the pellet (paw closure distance) for AAV9-UbiquitinWT- and AAV9-UbV.E4B-injected rats. Student's t test; n=3 AAV9-UbiquitinWT-injected rats; n=4 AAV9-UbV.E4B-injected rats. The data are presented as the means ± SEM in (C) and (D).

Table S1. Assessment of CRISPR Knockout Efficiency, Related to Figure 1, Figure 4 and Figure 5.

**Gene**	**Knockout efficiency***
UBE4B-sgRNA	82.0±1.5%
Pirh2-sgRNA	72.2±5.0%
Cop1-sgRNA	68.9±1.1%
HAUSP-sgRNA	70.3±2.6%
MDM4-sgRNA	79.9±2.4%
PTEN-sgRNA	79.3±4.4%
KLHL22-sgRNA	84.9±1.3%
P53-sgRNA	81.4±2.3%

*Control sgRNA or respective sgRNA vectors were cotransfected into HEK293T cells with Cas9 and the corresponding gene overexpression vectors. Cells were harvested 72 h after transfection, and total RNA was extracted. The transcript levels of the corresponding genes were measured using qRT–PCR with specific primers. The percentages shown were calculated from three independent qRT–PCR measurements of unchanged mRNA.

Table S2. Sequences of sgRNAs, Related to Figure 1, Figure 4 and Figure 5.

Gene	Sequence of the sgRNA
UBE4B	sgRNA1：AACTGACGCCTTCGCTGCTC
	sgRNA2：ATCTCTGTCGCGGTCCTTCC
	sgRNA3：TTCCCGAATCCACATCCACC
	sgRNA4：CCTGTGTAGGAGTTGCCCAT
	sgRNA5：CTCTTCTGTAGATTCGACGG
Pirh2	sgRNA1：TGCCTAACCACGAATCTTCG
	sgRNA2：GCTAGATCGTTTCAAAGTCA
	sgRNA3：GTGACAAGCTTTATACCTGC
	sgRNA4：GAATTGTCCAATATGCTTGG
	sgRNA5：TACAGTCTTCACAAGTCTGC
Cop1	sgRNA1：ATACGGTGCTACTCACTGAG
	sgRNA2：GATGGTCTTACCAAAAGCTG
	sgRNA3：AAAGACTCACCCAAGAAATT
	sgRNA4：CAAAATGTGGCCACAGCTTT
	sgRNA5：CAAGTGTATTCATCAGAGTT
HAUSP	sgRNA1：CAAAAACTTACGCAACTCCA
	sgRNA2：AGAACAAGTGGCTGATTCGC
	sgRNA3：TCTTCAGCACTGCTTGTGCG
	sgRNA4：TGCCTGTACAAAAACTTCAA
	sgRNA5：AGACACCAGTTGGCGCTCCG
MDM4	sgRNA1：TCCAAGTCAAGACCGACTGA
	sgRNA2：ACATCAGCTTCTATTAACAC
	sgRNA3：ATGTAGGTAATGCACTATCT
	sgRNA4：ACCTTCAGTCGGTCTTGACT
	sgRNA5：GAAGCAGCTCTATGATCAAC
PTEN	sgRNA1：ACCGCCAAATTTAACTGCAG
	sgRNA2：GCAGCAATTCACTGTAAAGC
	sgRNA3：TGTCATCTTCACTTAGCCAT
	sgRNA4：ACAATATTGATGATGTAGTA
	sgRNA5：AATCCCATAGCAATAATATT
KLHL22	sgRNA1：CTCATTCCGGTGGTACATAA
	sgRNA2：ACAGTTGTACATCTCGTCAG
P53	sgRNA1：TGAGGGCTTACCATCACCAT
	sgRNA2：ATAAGCCTGAAAATGTCTCC
Control sgRNA	sgRNA：GCGTCGTGACTGGGAAAACCC

Table S3. Sequences of UbV.E4B and UbiquitinWT, Related to Figure 6, Figure 7 and Figure S7.

Gene	Sequence
UbV.E4B	ATGTGGATTTTTGTTCAGACCCTGATGCGTAACACCATTACCCTGGAAGT GGAACCGAGCGATACCATCGAAAATGTTAAAGCAAAAATCCAGGATAAAG AGGGTATTCCGCCGGATCAGCAGTTTCTGATTTTTACCGGTACCCTGCTG GAAGACGGCCGTACACTGAGCGATTATAATATTCAGAAAGAAAGTCTGCT GCGCCTGGTTTGGCGTCCGCGTGGCCCTCTGAATTAA
UbiquitinWT	CTTCAGATCTTCGTGAAGACTCTGACTGGTAAGACCATCACCCTCGAGG TGGAGCCCAGTGACACCATCGAGAATGTCAAGGCAAAGATCCAAGATAA GGAAGGCATTCCTCCTGATCAGCAGAGGTTGATCTTTGCCGGAAAACAG CTGGAAGATGGTCGTACCCTGTCTGACTACAACATCCAGAAAGAGTCCA CCTTGCACCTGGTGCTCCGTCTCAGAGGTGGGTGA
